# Racial issues in psychiatry: a thematic analysis of an initial health equity educational activity for medical students

**DOI:** 10.1186/s12910-025-01215-3

**Published:** 2025-04-28

**Authors:** Paige Pickerl, Tanya Sorrell, Mennefer Blue, Kamaria Patterson, Neeral Sheth, Sahara Givens

**Affiliations:** Department of Psychiatry and Behavioral Sciences, Rush University Medical Center, 1645 W Jackson Blvd Ste 302, Chicago Illinois, Chicago, IL 60612 United States of America

**Keywords:** Medical Student Education, Psychiatry, Psychiatry Education, equity, Medical Racism, Qualitative, Substance Use Treatment, DEI, Student Perspectives

## Abstract

**Introduction:**

Current research documents both the historical impact of racism in healthcare as well as studies piloting antiracist interventions as part of medical training to ameliorate its stigma, bias, and consequences in medicine. The purpose of this study was to qualitatively analyze the impact of a one session lecture surrounding racial issues in psychiatry on third-year medical students’ thoughts and reflections surrounding the content.

**Methodology:**

Remote methodologies were used to engage medical students in a lecture created by a major University’s Substance Use Disorder Center of Excellence to address the legacy of racial issues in psychiatry as well as present interventions. The team collected anonymous evaluations via anonymous chat submission after each lecture. Qualitative evaluation data were compiled from 108 students across 11 sessions over the course of a year. The team reviewed major and minor themes and synthesized following the Standards for Reporting Qualitative Research (SRQR) guidelines for qualitative reporting.

**Results:**

We identified the following five themes:1) appreciation and notes on the content itself; 2) how the information presented can impact future clinical care; 3) the interconnectedness of social determinants of health and racism; 4) recognizing power dynamics with patients; and 5) opportunities for future education.

**Conclusion:**

Information compiled both from participating students and the available literature can inform future education efforts to build opportunities for antiracist training in medical education.

**Supplementary Information:**

The online version contains supplementary material available at 10.1186/s12910-025-01215-3.

## Introduction

Globally 1 in 8 individuals experience a mental health diagnosis [1, 2]. Unfortunately, access to appropriate care and treatment vary widely for mental health due to factors like socioeconomic disadvantage, migration, ethno-racial discrimination, gender discrimination, LGBTQ discrimination, social isolation, community and physical environment impacts, or childhood adversity [3]. These non-medical factors, known as Social determinants of health (SDOH), play an essential role in mental health outcomes beyond psychopharmacological interventions [4]. When comparing developed countries’ general quality of healthcare across medical disciplines, it is evident the United States falls behind their global counterparts. Despite the United States allocating the highest percentage of its GDP to healthcare compared to other developed countries, general health outcomes are significantly lower than those of its counterparts [5, 6]. The average life expectancy in the United States is 77.5, compared to the global life expectancy of 82.0 [7]. With over 40 million Americans uninsured and underinsured, there are higher rates of dissatisfaction with the American healthcare system [8]. Internally to the U.S., health outcomes are widely varied based on race. Life expectancies of Black and Indigenous communities average 72.8 years, and 67.9 years, respectively, with worse healthcare outcomes for Black, Indigenous, and Hispanic populations [9]. This is exacerbated by lack of healthcare access, lower insurance access, and less preventative health measures available to Black, Indigenous, and People of Color (BIPOC) communities [10], across states.

Chicago, in particular, is a diverse city with the federal census data for 2024 estimating the most populated racial/ethnic groups being Non-Hispanic White (39.0%), Hispanic or Latino (29.6%), and Black (28.4%) individuals [11]. The distribution of these groups within Chicago is notably segregated, with the majority of Black and Hispanic/Latinos residents in the South and West sides, while White residents predominantly occupy the North and Upper West side of the city [12, 13]. For Chicago BIPOC communities in particular, the effects of systemic racism and its impact on access to healthcare are felt at a local level [14]. With an overall life expectancy score of 76.6 years, Chicago demonstrates a significant life expectancy gap of 11.6 years between Black and non-Black individuals, which is more than twice the national discrepancy of 5.7 years [15]. This disparity reflects the city’s historical legacy of segregation, particularly evident in its South and West Side communities, which experience lower levels of employment and household income. According to the Chicago Health Atlas hardship index, a composite score that incorporates unemployment, education, per capita income, the percentage of people living below the poverty level, overcrowded housing, and age dependency, the West and South Sides of Chicago display higher index scores compared to their more affluent neighbors to the north and east [16]. Furthermore, the southern and western regions also experience a number of health disparities, such as less access to care, which results in lower overall health status and higher mortality rates from cancer, diabetes, and drug use [17]. Residents of some West Side communities can expect to live up to 14 fewer years than residents of Chicago’s Loop, just 4.5 miles east [14]. Gaps in healthcare access are particularly pertinent regarding mental health outcomes for BIPOC individuals [18].

Although rates of psychiatric disorders are similar among racial/ethnic minorities and Whites, minorities are less likely to receive mental health services and more likely to receive poor quality care than White Americans [19, 20]. These disparities are in part explained by social and economic factors and a lack of engagement in general health care by African Americans and Latinos who are less likely than whites to have a regular primary care physician, an annual health care visit and are more likely to report emergency room use [21]. These factors contribute to the increase in the severity and persistence of mental health disorders among racial and ethnic minorities [22]. Today, Black Americans are still over diagnosed with schizophrenia or intellectual disabilities due to impersonal and culturally insensitive modern screening tools [23]. Likewise, African Americans and Hispanic/Latino individuals were more likely to perceive a lower quality of care and report dissatisfaction with their relationship with providers [18]. While there is a global standard of otherness that impacts mental health care for minority communities [24], the United States unique historical relationship to systemic racism must be addressed in psychiatric care.

Psychiatry was historically weaponized against Black individuals starting in the 19th century during enslavement. Enslaved Africans whose behavior was attributed to rebelliousness or escaped were diagnosed with Drapetomania, a disease “peculiar to Negros” [25]. The desire for freedom was characterized as a disease that must be treated and is an example of scientific attempts to justify racial hierarchy. Dr. Benjamin Rush, who is often referred to as the father of psychiatry; in 1797, claimed the color of black people’s skin was caused by a disease he called Negritude [26], with the “cure” involving muriatic acid baths, which promoted biases and stereotypes of black person’s skin that persist today [27, 28]. Medical history during the post-Civil War era was marked largely by a time when medical schools did not allow Black individuals to enroll, as well as the 1910 Flexner Report. This report resulted in the closing of all but two Black medical schools, subsequently leading to fewer healthcare options for Black patients [23]. Recommendations in the Flexner Report also fueled persistent and pervasive racist ideologies in predominantly white classrooms and did little to challenge racist and unethical medical practices. Additionally, Black physicians were excluded from the American Psychiatric Association (APA), which allowed racist biases in psychiatric practices to persist [23].

Although major criticisms were made in 1969 it was not until 2021 that the APA apologized to People of Color for its role in medical racism; in addition, they acknowledged the organization’s sustainment of racial hierarchical theories which contributed to the racial disparities we still see today [29]. Shim (2021) states that interventions are needed not only to address existing health disparities and misconceptions, but also the trauma caused by racial injustice, which has considerable effects on the mental health of minoritized populations. This issue was highlighted more recently during the COVID-19 pandemic and after the murder of George Floyd. Specifically, “examples of racial injustice (including COVID-19 inequities, police killings of Black people, and mass shootings targeting specific racial and ethnic groups) are prevalent in U.S. society. There is a growing understanding that trauma caused by racial injustice has extensive impacts on mental health” [30]. The influence of medical racism must be acknowledged as we work to address the racial inequalities evident in psychiatry.

Few studies have been done to further understand how medical students integrate SDOH into early treatment practices. One team found students’ will differentiate treatment approaches based on the amount of social context they receive about a patient [31]. Studies documenting the results of educational interventions to address health disparities by race for medical students report positive results in student preparedness and activation. For example, DallaPiazza et al. (2018) conducted a mandatory evaluative intervention with medical students during the academic years of 2016–2019. From this intervention, developed in conjunction with faculty and staff, the authors concluded that “Active student involvement in curriculum development and small-group facilitation was critical for successful buy-in from students”. The authors added that “additional content on bias, stereotyping, and health care disparities will be the focus of faculty development programs will also be integrated into the clerkships to build on these important topics as students are immersed in clinical care” [32]. Similarly, Hess et al. (2020) launched 4 phases of institutional change implemented by activism from medical students. After the study, the authors reflected on lessons learned, personally and structurally, and on several challenges. On a personal level, they highlighted the need for humility, trust, and curiosity as crucial tenants for building antiracist training. On a structural level, they noted the importance of using correct language and looking at smaller issues as something connected to a larger problem. The biggest challenges they reported were shifting individuals’ mindsets, managing resistance to change, and working smarter [33]. Lastly, a 2018 study by White-Davis et al., “Addressing Racism in Modern Medicine,” sought to understand attitudes and perceptions of teachers of family medicine through an interactive workshop. The 90-minute workshop included a 3-minute video depicting racial and gender micro-aggression within a hospital setting. The authors found that “qualitative information from small group facilitators and large group discussions identified some participants’ emotional reactions to the video [shown to participants], including dismay, anger, fear, and shame. A pre/post survey (N = 72) revealed significant changes in attitude and knowledge regarding issues of racism and in participants’ personal commitment to address them…. Findings also suggest this workshop improved confidence in teaching learners to reduce racism in patient care” [34].

Medical education interventions concentrating on antiracist training show necessary educational models for student preparedness. Several emerging studies indicate that specific anti racist training is required in psychiatry in light of psychiatry’s racist historical context, noted above. Hansen et al. (2018) highlighted 3 core tenants that are crucial for integrating psychiatry education and antiracist education. These are “ understanding patients’ experiences of illness in the context of structural factors…, intervening to address structural factors at institutional levels…, and developing community connectivity and structural humility, a posture of collaboration with community leaders and with other disciplines and of patience with the slow pace of structural change” [35]. Additionally, Shim (2021) charges readers to dismantle structural racism in psychiatry through continuing education and self-reflection on racism in medicine, changing social norms and systems, and addressing public policies that affect patients [30]. Another research team is currently conducting a study titled “Dismantling Structural Racism in Psychiatric Residency Training: Nurturing a New Generation of Black, Indigenous, and People of Color (BIPOC) Psychiatrists” [36]. Although their research focuses on how to dismantle racism in the classroom, particularly with regard to psychiatric residencies, further research is needed in this area. As noted by third-year medical students in a Letter to the Editor at Johns Hopkins in the wake of the death of Freddie Gray, “We need to expand our lessons beyond awareness of health inequity: Medical schools must develop, longitudinally reinforce, and evaluate skills that will equip their graduates to combat racism and structural oppression. Furthermore, competency in these areas should be enforced as thoughtfully and rigorously as our traditional clinical training is” [37].

Through Social Determinants of Health and other frameworks, evidence presents that race and community racism have a functional impact on an individual’s physical and mental health, as well as their health outcomes and subsequent relationships with their health care systems [38]. There is a precedence in current literature which surrounds both the historical impact of racism in health care as well as current interventions being done in order to ameliorate the stigma, bias, and consequences of racism in the medical community. Globally, the call for antiracist training has been noted by The Canadian Medical Education Directives for Specialists (CanMEDS) as a foundational core competency [39]. Through the suggestions of the Asian Journal of Psychiatry, they note the necessity of incorporating sociological frameworks and cultural competencies into graduate psychiatric education [40]. Educating American medical students on the historical precedence of racism in health care and medical education while also addressing current social and structural issues in psychiatry is important in Rush’s commitment to improving health disparities in Chicago’s West Side.

In response to the need for interventions to address racism in medicine, particularly in the field of psychiatry, a training session was developed in the fall of 2020 by the Rush University Medical Center in conjunction with an associate professor of psychiatry, the Director of Clinical Education Graduate Medical Education Committee. The contents of the lecture were informed by an extensive literature review which analyzed the historical contexts and impact of racism in medicine as well as current interventions to ameliorate its stigma, bias, and consequences. Results of this evaluation are from student feedback from the lecture.

## Methodology

Through the lens of a Social Determinants of Health (SDOH) framework, the topics presented for this intervention highlighted the functional impact of race and community racism on an individual’s physical and mental health, as well as their health outcomes and subsequent relationships with their health care systems [38]. Lecture activities comprised a Q&A session, case studies, and discussions. Topics included historical contexts of racism in psychiatry, and present health inequalities resulting from outdated healthcare practices, all essential for training medical students in order to address racism in medicine. Initially this content was presented to third-year medical students during the last week of their 4-week psychiatry rotation in the form of a 60-minute didactic and 60-minute discussion. Based on student evaluations requesting the content earlier in their rotation, the lecture was moved to week 1 of the practicum in July 2022. Concurrently, a 30-minute introduction of the lecture was added to first-year medical students’ curriculum.

### Literature review for course curriculum

In order to provide full historical context for students, the team began a two-pronged literature review in order to inform the intervention. The first being on the historical context of racism and the field of psychiatry, and the second being on classroom interventions for training medical students. The course began by highlighting psychiatry’s long history of racism and its origins in slavery, when behaviors from enslaved Africans were pathologized. The lecture also highlighted examples of Black people as experimental subjects, generational and historical trauma, and early ideas of eugenics. The secondary literature review was necessary for educators to gain an understanding on current classroom-based, anti-racist training interventions for medical students. By understanding how American medical history has contributed to systematic racism and continuing healthcare inequities, today’s medical practitioners can more effectively construct interventions for both community healthcare settings and medical classrooms. Ideally, these interventions will lessen the racial gap in healthcare services, particularly in psychiatry. They will also help to build a more culturally competent workforce, which may lead to a decrease in over diagnosis of Black patients, particularly for schizophrenia or intellectual disabilities.The literature analysis provided an understanding of the history of racism in psychiatry and current interventions available to students; thereby providing the team an informed foundation for developing the lecture.

### Curricula development

The Director of Graduate Medical Education committee in partnership with an Associate Professor under the Psychiatry department and their educational coordinator developed the curriculum beginning in November 2020. Monthly meetings were conducted to review content and revisions, per the literature analyses conducted. The curriculum was approved in the spring of 2021 by the Graduate Medical Education Committee, and initiated with third year medical students during their psychiatry rotation, beginning May of 2021. Every student in their third year psychiatry rotation was presented the lecture. Data on the program was collected August 2021 until January 2023. The team completed an initial 6 month review of the curriculum, objectives, outcomes, and initial assessments in the event revisions to curriculum were necessary. All content presented was scripted, allowing for presenters to have consistency.

Learning objectives for the course were as follows:

1. Identify/understand the historical context and foundations of racism, globally and in the United States.
2. Describe current structural and institutional racism issues in psychiatry and how they have impacted persons of color.
3. Apply cultural and structural competence and knowledge of intersectionality to/in diagnosis and treatment of mental illnesses and substance use disorders.
4. Recognize ongoing efforts to address individual to structural racism issues, and the ongoing pushback from some groups.

The lecture was delivered by a guest lecturer from the Psychiatry department by either the associate professor or education coordinator. It began with a Q&A around stigmatizing beliefs that still exist in psychiatric care, which created a space for students to unpack this topic in a group setting. The presentation went on to discuss the history of racism in psychiatry, as was highlighted above, specifically focusing on psychiatry and eugenics, phrenology, and the exclusion of Black doctors. The slides presented during the Racial Issues in Medicine learning activity are attached in appendix 1.

The presentation then informs on SDOH and their relationship to racism in healthcare. The last section of the lesson focused on where healthcare is presently working to combat racism, particularly in psychiatry. The discussion on current healthcare also highlighted growing concerns that Black physicians and medical students are leaving psychiatry due to racial issues experienced in the field [41]. During this portion of the lesson, students worked through several case studies in a group session to navigate individual, institutional, and structural racism. Case studies are also provided in slides within appendix 1.

The goal of case discussions was to allow for implicit bias admission in a non-threatening supportive manner for students. This activity included content related to diagnosis of schizophrenia, accented voices, calls to security, and how those issues negatively impact and are more likely to occur with minority clients. Once the class was complete, students submitted their review and thoughts about the session anonymously to the presenter via the private chat option for the virtual classroom. Open ended course evaluation for this study asked students to reflect on the training they just received and how it can impact their patient care. The question is presented in appendix 1 and was written by content developers. The questions asked orally of the students during the discussion were: (1) What was your overall satisfaction of content presented?; (2) How did you feel the information may be incorporated into your practice? And; (3) What is any additional feedback on how we can improve content for future participants?

### Qualitative analyses

Tenants of Standards for Reporting Qualitative Research (SRQR), a 21-item guideline for reporting standards, were followed in order to systematically compile qualitative themes and adhere to standardized practice for an ethnographic analysis of year 3 medical students. SRQR specifically notes the necessity of justifying the choices made in building research design and methodology in order to promote transparency in decision making [42].

The data were collected by the presenter and anonymized for further decontextualization for the research manager, who did not partake in delivering the lecture. She completed a first round of coding and created candidate themes. Once this initial analysis was complete, the Principal Investigator reviewed the themes in order to triangulate results and findings. SRQR Checklist can be found in Appendix 2. Coding was concurrent with the lectures so as to determine when the appropriate saturation had been met to begin analyzing the data. Additionally, the presentation had been in use for a full year, and if any revisions were needed to the presentation, reviewing the information gathered would be beneficial when making those changes. Major and minor themes were reviewed and synthesized by first coding for information about the content of the course itself and for information relating to personal reflection.

## Results/Themes

### Participants

252 students attended the lecture from August 2021 to January 2023 over 11 sessions. Student demographics are as follows: 52% (*n* = 131) male, 48% (*n* = 121) female; By race, 52% (*n* = 132) White; 23% (*n* = 57) Asian; 10% (*n* = 25) Hispanic/Latino; 7% (*n* = 17) unknown; 6% (*n* = 14) Black/African American; 3% (*n* = 7) non-Hispanic multi-race. Of those students, 108 individuals submitted answers to the evaluation questions. Submission responses were anonymous, therefore the gender and racial makeup of the students who submitted responses is unknown. Questions students answered comprise:

1. What was your overall satisfaction with the content presented?
2. How did you feel the information may be incorporated into your practice?
3. What is any additional feedback on how we can improve content for future participants?

### Themes

Results were categorized into three major themes and one minor, each detailed below.

#### Appreciation/Notes for content: (*n* = 52)

Approximately 48% of students expressed general appreciation for the content itself, appreciation for the flow and discussion in the class setting, or shared notes for strengthening the presentation in the future. One student expressed, “I really appreciate that discussion of racism and social determinants of health are included in the curriculum. I like how this session was interactive and included real [life] scenarios as this helped make the session even more relatable. I will definitely use what I learned today moving forward! Thanks.” Another student noted, “It was helpful reviewing structural issues that can impede on giving proper healthcare. It’s always helpful to discuss scenarios since there is always going to be a situation that you are unfamiliar with or unaware that you won’t be prepared for.” Similarly, another student shared, “This was a very well done module, I appreciated the opportunity to review some of the historical info in the video posted and then having the entirety of the session be focused on discussion with my peers regarding this topic. The cases were great visualizations for how frequently we encounter our own biases in medicine, especially in regard to race and how we perceive our patients.”

There were a handful of notes on content improvement that will be taken into account. When discussing appreciation for the flow of the content with a note for improvement, a student noted: “Really appreciate the case-report style discussion. I would have liked to spend more time on the 3rd case report and be able to think about what we as students and future residents can do for de-escalation techniques without security, since we only briefly touched on that.”

One minor theme that should be explored deeper centered around students considering how these presentations offered opportunities for continuing their education in a diverse setting, as well as a responsibility to continue unpacking racism in their education. One student wrote, “I think one reflection I have on my experience at Rush, in general, is that we are very privileged to have the kind of diverse patient population that we see. Coupled with sessions like this, I think it gives us the tools to be better clinicians. It is our responsibility to take the opportunities that we have to continuously educate ourselves about racism and how it intersects with health care. It is important not to assume, but to listen.”

#### How racism impacts clinical care directly/next steps in clinical care: (*n* = 36)

The second most prominent point, comprising 33.3% of students, centered around reflections on how racism directly impacts clinical care and what they can do in a patient setting to recognize biases and support patients in real time. One student noted: “While racism is prevalent in all areas of medicine, it’s especially important to recognize in psychiatry since it could be a contributor to our patients’ presentation. In recognizing this, it’s also important to acknowledge that we are not perfect as providers and it can be impactful to ask for help in how to address racism/racial inequity, even if from our patients themselves. As providers that do not identify as a racial minority, we have to make a special effort to understand our patients’ experiences and to walk in their shoes.” Another student noted, “I really enjoyed this session. I liked the use of case studies as a way to discuss issues related to racism in psychiatry and medicine in general. It put the issues into perspective, going forward I hope to think about my own implicit biases when I see a patient so that I can avoid pre-judging patients and provide equitable care to all.” When reflecting on the case examples, one student noted “In the first case it [discussed] the importance of communication with the office staff and the provider when patients have setbacks such as being late. It is vital to not send patients away because we don’t know what barriers to care they may be facing and we need to make sure we know and are flexible.”

#### Interconnectedness of SDOH, racism and health outcomes: (*n* = 7)

The third theme, brought up by 6.5% of students, revolved around reflecting on the larger patterns of interconnectedness of SDOH and racism in medicine. For context, SDOH is defined as “the conditions in the environments where people are born, live, learn, work, play, worship, and age that affect a wide range of health, functioning, and quality-of-life outcomes and risks” [38]. These seven students reflected on how that synthesizes into practice. One student noted, “As medical students recently finished with our preclerkship studies, I feel it is easy to fall into the trap of looking at the issues our patients face solely through a biological/physiological lens. It is important that we do not neglect to consider the social context in which our patients live as we cannot expect to fully address our patients’ needs if we do not tailor our approach in such a way that is amenable to our patients. Racism, or discrimination against any minority for that matter, is a real and important contributor to stress and mental health issues in today’s society and we must at least recognize that those different from ourselves live under different circumstances and face unique challenges.” Another student noted, “The interconnectedness of minority statuses and inequities is truly staggering. To be black or brown, experience poverty, have unstable housing, have decreased access to medical and mental health care, and to be incarcerated are all part of a single web.”

#### Recognizing power dynamics: (*n* = 6)

A handful of students (5.5%) contributed to one minor theme that emerged around how they will recognize power dynamics in their active practice. One student wrote, “And beyond simply providing more services, we must provide quality services. The lecture speaks to the power the medical institution has to pathologize, stigmatize, and inadequately treat our patients. We must always be cognizant of this dynamic, because our patients are still suffering under the abuse of this power. We’ve seen this come up recently in our discussions on the hospital’s use of race-based GFR calculations. Given the magnitude and quantity of these issues, it can all seem daunting, but I’m hopeful that through our commitment change will come.” Another student noted, “When discussing topics such as race within psychiatry, we must remember first to listen and validate our patients’ concerns. Listening to what our patients have to say is the first step to a better understanding where our patients come from and how we can try to improve upon their previous experiences.”

## Discussion

This study used qualitative analysis to review the impact of a lecture surrounding racial issues in psychiatry on third-year medical students between August 2021 to January 2023. Students reported an overall positive and enlightening experience in receiving the lecture and engaging in a discussion of case studies. Many of them expressed appreciation for the content and format. Many students elaborated on how this lecture will impact their clinical care moving forward. Others shared how the content influenced their thinking on the role of SDOH in health outcomes and on recognizing power dynamics in a health care setting. Team also noted minor themes of course improvement from students, in order to inform edits to curriculum in future lectures. Future themes in building curriculum with a guided framework allows educators to build antiracist training materials for their students.

Many institutional bodies have provided recommendations in order to begin classroom interventions navigating inequity in psychiatry [24]. Bughra et al. recommends based on their review that globally educators should ensure students practice communication skills, learn to understand illness from a social determinants of health framework that centers in a multidisciplinary approach, and learn to manage stigma and discrimination [40]. The three components of their recommendations were reflected by the students throughout the emerging themes. First, student communication skills were practiced and reinforced through case based learning. Cased based learning has been a longstanding pedagogical standard in order to engage adult education involvement and knowledge application [43, 44]. It has also been used successfully in a broader context to navigate racism in healthcare [45]. Specifically, when the students noted their appreciation for the content many of them made reference to the case examples as being a particular point of the presentation they found helpful. Beyond their appreciation for the case, when the students reflected on their next steps in clinical care and practice, they noted the cases as something that will support the future impact of their work.

Second, their recommendation requires that students learn and understand illness from a social determinants of health framework and a multidisciplinary approach. In the minor theme on Interconnectedness of SDOH, Racism, and Health Outcomes, students pointed to the necessity of looking beyond biological aspects of treatment in order to understand the social contexts in which they live. Applying sociological frameworks to medicine through providing historical contexts, case examples, and modern issues in medicine allow us to see that even one-time interventions allow students to reflect on larger multidisciplinary integrations into medicine [31]. Other researchers also “found that…clinicians’ causal beliefs may influence their choice of treatment options.” [46] Lastly, Bughra et al. emphasizes the need for students to manage stigma and discrimination. This competency was interwoven across all themes recognized from students. While some students highlighted the cases as a basis for initially recognizing encounters in racism, the final minor theme emphasized students’ recognition of power dynamics involved in medicine. Recognizing power dynamics in medicine, particularly in the field of psychiatry, is crucially important. Without this recognition, coercive treatment and power imbalances can persist [47].

In parallel with Bughra’s review on educational necessities, Cenat et al.'s team highlighted four key components from the educators perspective on creating antiracist training measures that exist in their scoping review: 1. Understand “the cultural, social, and historical context of mental health problems” 2. Develop “awareness on self-identity and privilege” 3. “Recognize oppressive behaviors” and 4. Adopt “antiracist competencies in therapies and alternative approaches” [48]. All four key components for education were provided in the lecture. These can be reviewed in the literary analysis or Appendix 1. Importantly, as this intervention was created and conducted in Chicago with real cases from Rush University, replicating this education in different community areas across the country, or globally, may require localized systematic reviews. In other areas of the US for example, creating education on holistic mental health interventions to address historical trauma for indigenous communities [49], or the need for trauma informed care in Muslim communities [50], would be necessary. In both instances, Cenat et al.’s four components of building antiracist training measures can provide the framework to build psychiatric education.

Both research teams provide frameworks on anti-racist education in medicine. Bughra et al.’s team provided three key components of which students’ knowledge building can be measured from the curriculum. In contrast, Cenat et al. provides four measures of framework educators can adopt in order to build successful training modules. Several classroom interventions have emerged from the desire to build antiracist education in healthcare. Utilizing this presentation as an initial intervention with students to then build continuing education for residents of psychiatry could support in building future interventions. Future intervention research, particularly in residency, is needed.

### Limitations

There were several limitations in this body of work. As a pilot program at one university, reflections from students were not collected across multiple institutions. Therefore, students may have a different baseline of knowledge about racism in the context of medicine. Future studies should explore a multi-site approach that incorporates standardized racial sensitivity measures to produce more objective outcomes. Furthermore, it would be beneficial to follow students throughout their medical training and collect post-test data at 6 and 12 months of their clinicals to explore any longevity in retaining the content presented during this lecture. It is necessary to see if the qualitative information is generalizable across student bodies. Additionally, the data were collected through open forum, and not through personal interviews. Therefore, researchers were unable to obtain demographic information of the individuals who responded, outside of the general demographics of individuals who attended the lecture. Personal interviews with the students may have provided more robust thorough responses regarding the presentation, as well as precise demographic information. Third, no longitudinal data were collected from the students. While gaining their initial impressions on the curriculum was important, seeing how the curriculum impacted their overall care during rotations would have provided further exploration on patient outcomes.

## Conclusion

Few educational presentations for medical students thus far have focused specifically on the correlation between racism and the field of psychiatry once students begin residency, where disparities in mental health care access, diagnosis, and treatment remain a notable problem [51, 52]. This is particularly crucial for Black patients, who have long been discriminated against in healthcare practices, targeted by unethical research practices, and neglected by the presence of long-standing pseudoscience. Medical education should continue integrating historical and sociological frameworks across all areas of medicine as an initial intervention. Information compiled both from participating students and the available literature can inform future education efforts to build antiracist training in medical education. Training medical students to understand the historical context of inequitable healthcare can support in building future health interventions for BIPOC patients in order to ensure both providers and patients have the tools they need for successful health outcomes. Future research is encouraged to include qualitative interviews with students overtime to provide in depth results for building education interventions on antiracism.

## Electronic supplementary material

Below is the link to the electronic supplementary material.

Supplementary Material 1

Supplementary Material 2

Supplementary Material 3

## Data Availability

The datasets used and/or analysed during the current study are available from the corresponding author on reasonable request.
