# Supplementary material for: Racial issues in psychiatry: a thematic analysis of an initial health equity educational activity for medical students
Source: BMC Med Ethics. 2025 Apr 28;26:53. doi: 10.1186/s12910-025-01215-3 (PMC12039112; doi:10.1186/s12910-025-01215-3)
Supplement: Supplementary file 2 — Supplementary Material 2 [file 12910_2025_1215_MOESM2_ESM.docx]

Open Ended Question to Students

1. “Please Include your thoughts and reflections on today’s training and how it can make an impact on your care of patients during and after your rotation.
