# Supplementary material for: Racial issues in psychiatry: a thematic analysis of an initial health equity educational activity for medical students
Source: BMC Med Ethics. 2025 Apr 28;26:53. doi: 10.1186/s12910-025-01215-3 (PMC12039112; doi:10.1186/s12910-025-01215-3)
Supplement: Supplementary file 3 — Supplementary Material 3 [file 12910_2025_1215_MOESM3_ESM.pdf]

Racial issues in Psychiatry: From Hate to Hope

Tanya R. Sorrell, PhD, PMHNP-BC
Paige Pickerl, LCSW
Mennefer Blue, B.A

https://www.kevinmd.com/blog/2021/07/heal-the-system-medicine-must-be-anti-racist.html
As part of a social justice and restorative curriculum, we have to discuss the truth in medicine and our specialties in understanding how we've gotten to this point, and to learn how to move forward for all of us.

Lopez, S., Sotto, S., Lakshmin, P., Skinner, D., Marshall, A. L., Zhang, S., Pho, K., Abramson, S., Badsha, H., Tamaren, J., Naumovski, Z., Shamim, Dr. H., Villagra, M., Rigert, J., Stombaugh, L., Bingham, D., Lazarus, A., Grand-Gassaway, A., Galinsky, D., … Famuyiwa, O. (2021, July 21). *Heal the system: Medicine must be anti-racist*. KevinMD.com. https://www.kevinmd.com/blog/2021/07/heal-the-system-medicine-must-be-anti-racist.html

Learning Objectives

1. Identify/understand the historical context and foundations of racism, globally and in the US
2. Describe current structural and institutional racism issues in psychiatry and how it’s impacted persons of color
3. Apply cultural and structural competence and knowledge of intersectionality to/in diagnosis and treatment of mental illnesses and substance use disorders
4. Recognize ongoing efforts to address individual to structural racism issues, and the ongoing pushback from some groups

Benjamin Rush, MD (1745-1813)

“The physician who considers every different affection of the different systems of the body … as distinct diseases when they arise from one cause, resembles the Indian or African savage, who considers water, dew, ice, frost and snow as distinct essences.”

- Early theories were Blacks immune to mental ills
- His diagnostic theory of “**Negritude,**” which considered melanation to be similar to a mild form of leprosy, and the cure was to become white. The cure- *muriatic acid baths*.
- His trainee- Samuel Cartwright- “**Drapetomania**-” Blacks near free states went psychotic- ran away
- Created policies that have further marginalize minority groups - felt Indigenous peoples should be eliminated
- Later in life, co-founded anti-slavery society and attributed the insanity of negritude to enslavement

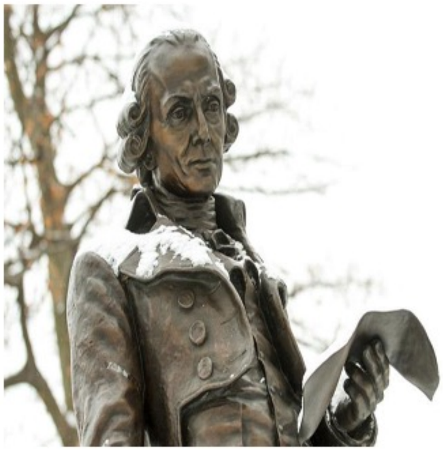

1. Historical Context
    1. Origins of Racism in American Medicine and Psychiatry
    2. Psychopathology of the “White Man’s Burden”

North R. L. (2000). Benjamin Rush, MD: assassin or beloved healer?. *Proceedings (Baylor University. Medical Center)*, *13*(1), 45–49. https://doi.org/10.1080/08998280.2000.11927641

King LS. The Medical World of the Eighteenth Century. Chicago: University of Chicago Press; 1958. pp. 223–224.

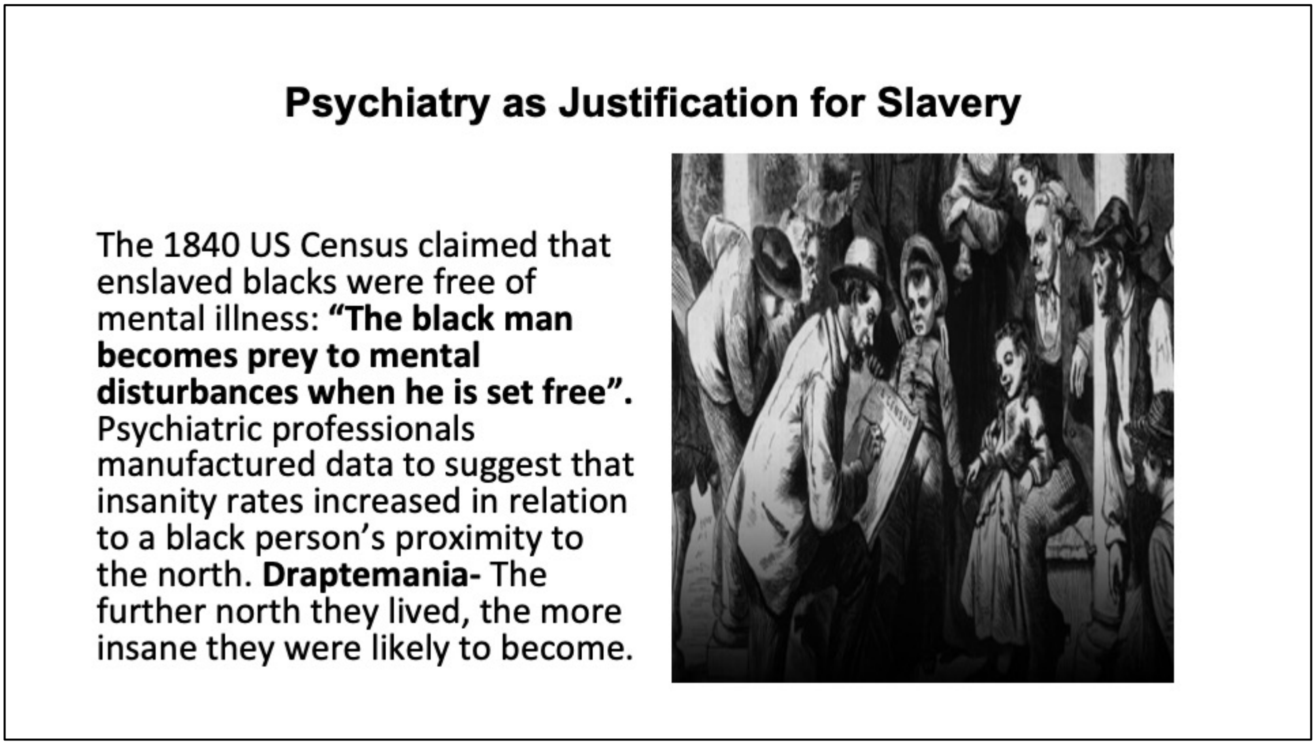

It was accepted by many psychologists that members of the African race had smaller brains, as well as a more natural instinct for labor, and were “psychologically adolescent” compared to members of the European race (1). “Drapetomania” was the term used for the supposed mental illness that caused Africans to flee captivity (2).

the Tuskegee syphilis experiment

mistrust and underrepresentation of African Americans in medical research

‘generalizability’ of white males to the population

Image Source: *Lewes lunch and learn on November 4*. The Quiet Resorts. (2016, November 3). https://thequietresorts.com/lewes-lunch-learn-november-4/

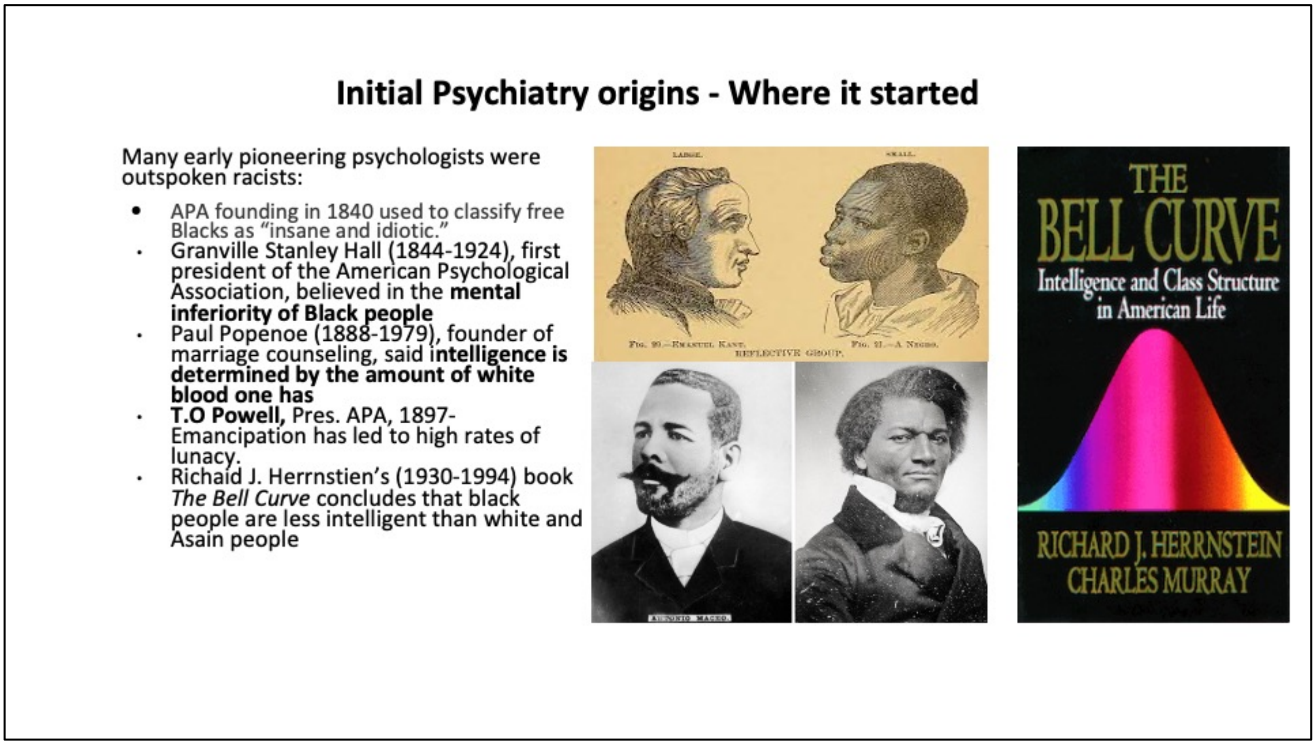

Dismantle Racism in Psychiatry & Society
https://www.psychiatrictimes.com/view/dismantle-racism-in-psychiatry-society
Moffic, S., Bailey, R. K., Clark, F. A., Cohen, C., Friedman, C., Isom, J., & Malhortra, A. (2020, August 11). *Dismantle racism in psychiatry & society*. Psychiatric Times. https://www.psychiatrictimes.com/view/dismantle-racism-in-psychiatry-society

Antonio Maceo- founder of Puerto Rico, Fredrick Douglas- both with white mothers

- Ktitowsky. (2017, March 5). *Phrenology and "Scientific Racism" in the 19th Century*. Real archaeology. https://pages.vassar.edu/realarchaeology/2017/03/05/phrenology-and-scientific-racism-in-the-19th-century/
- Black, G. (2020, October 12). *The whitewashing of black genius*. Scientific American. https://www.scientificamerican.com/article/the-whitewashing-of-black-genius/

Poskett, J. (2016). PHRENOLOGY, CORRESPONDENCE, AND THE GLOBAL POLITICS OF REFORM, 1815–1848*. *The Historical Journal, 60*, 409 - 442.

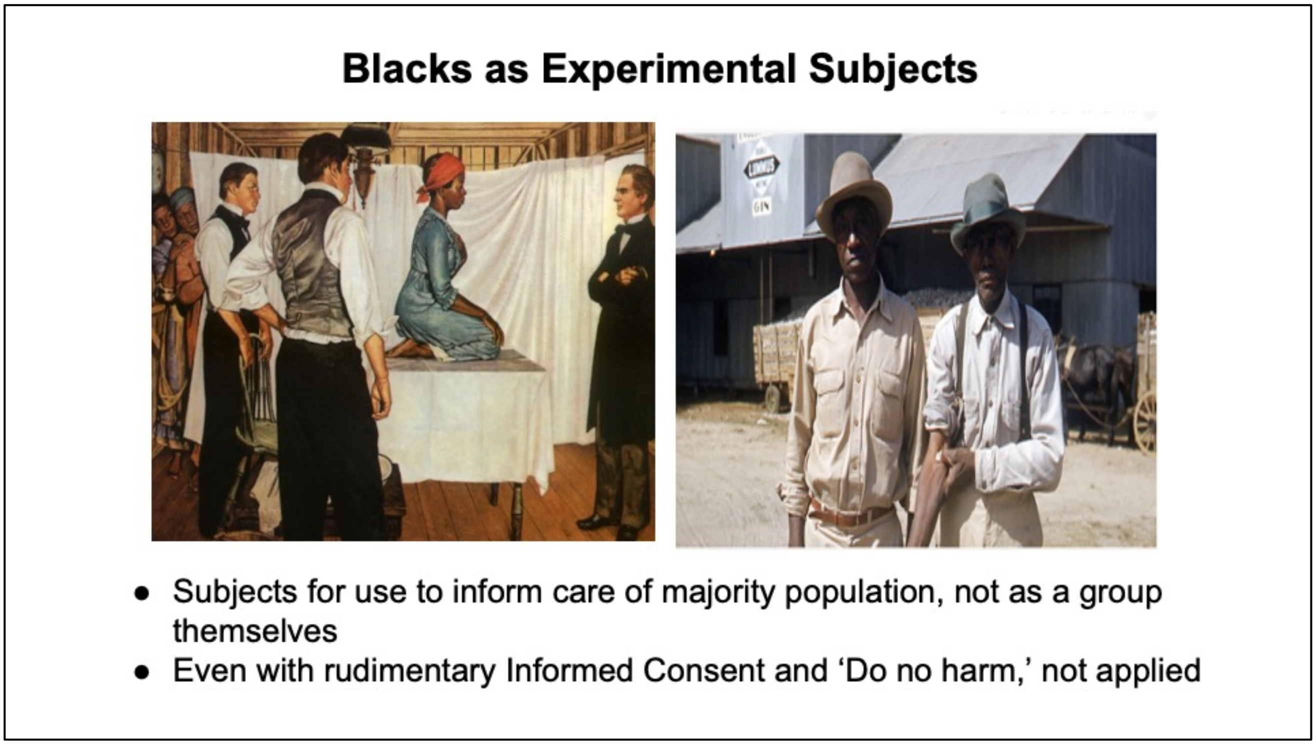

As property and non-citizens, use of enslaved in development of medicine, war techniques, was profound, with enslaved as victims.

- Downes, H. (2020, October 20). *Honouring the slaves experimented on by the “father of gynaecology.”* The Conversation. https://theconversation.com/honouring-the-slaves-experimented-on-by-the-father-of-gynaecology-148273

https://eji.org/news/history-racial-injustice-tuskegee-syphilis-experiment/
(Equal Justice Initiative, 2020)

But there are so many, before Tuskeegee (the formation of the speciality of OB/Gyn was operations on enslaved Black women,Henrietta Lacks in 1953- HeLa cells, after - 2010- Havasupi tribe sued Arizona State for misusing their genetic samples without permission

Trails of Tears, and Historical Trauma on Native Americans

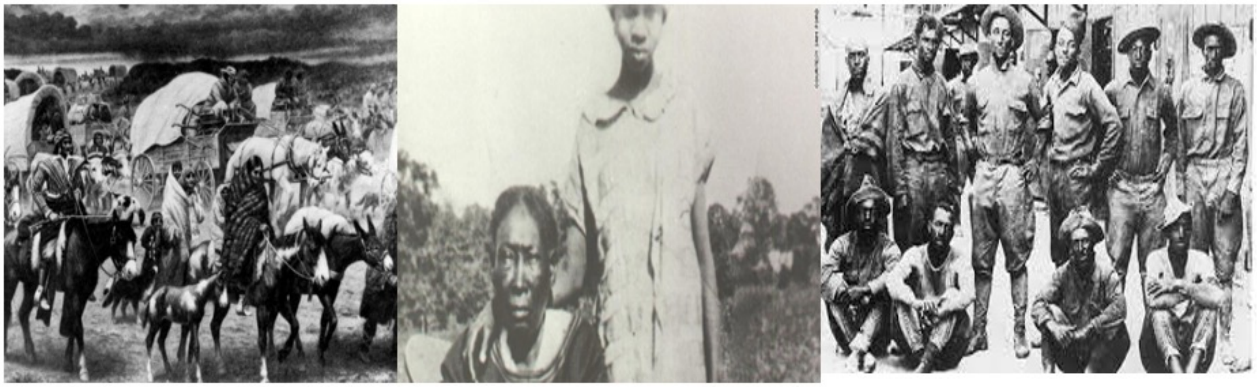

- Manifest destiny
- Black shared enslavement, assimilation, or Native American annihilation
- Buffalo Soldiers (10%) and Cowboys (20-25%)

1. Manifest Destiny and the Native American Genocide
    1. Death, Trails of tears, and historical trauma on Native Americans
        - Why weren't Natives enslaved as Africans were? They were- https://www.npr.org/2017/11/20/565410514/an-american-secret-the-untold-story-of-native-american-enslavement
        - We say that there have been 12.5 million Africans forcibly transported across the Atlantic as slaves into the New World. That is a very powerful thing to say. And I wanted to get a rough sense of how Indian slavery compared to that. So I came up with a figure of 2.5 to 5 million Native Americans enslaved throughout the Americas since Columbus to 1900.
        - It's a misnomer to think enslaved Africans were receptive and didn't mutiny against slavery, those enslaved that ran away were punished, mutilated or killed. The multiple languages of enslaved Africans made wholesale mutiny attempts problematic- see Haiti who were able to end Slavery, but had to pay

‘reparations’ to France for their freedom through 2015- this isn’t factored into discussions as to why Haiti has had difficulties since their freedom- other countries shunned/worsened Haiti’s ability to grow

2. The Indian School and Trauma, abuse, and aculturing
3. Native Americans and alcohol use
4. Benjamin Rush and thinkers of the day thought “Negros can be assimilated (him through muriatic acid) but that Indigenous should be eliminated.
5. Cohen, R., Penman, M., Boyle, T., & Vedantam, S. (2017, November 21). *An American secret: The untold story of native american enslavement*. NPR. https://www.npr.org/2017/11/20/565410514/an-american-secret-the-untold-story-of-native-american-enslavement

Civil War and Early Freedom

Highest signees to any War-

- 70% of free Black men served in Union Civil War (10% of entire army)
- Blacks moved when able to cities and developed separate economies
- South quickly had educated Blacks in government and Congress

Black Codes → Jim Crow

- T.O. Powell's theories used to incarcerate Blacks for crimeless behavior
  - Loitering, not working, taking a non-slave type job (servant/labor)
  - Political rise in Black communities met with massacres
  - Black Asylums for those termed "insane." "treatment" - hard labor
  - Black men threatened by Whites escaped to the West- cowboys- Buffalo soldiers
  - Codes limited political and economic aspirations of Black communities
  - VIolence and rise of Klan eliminated competitive, growing Black communities

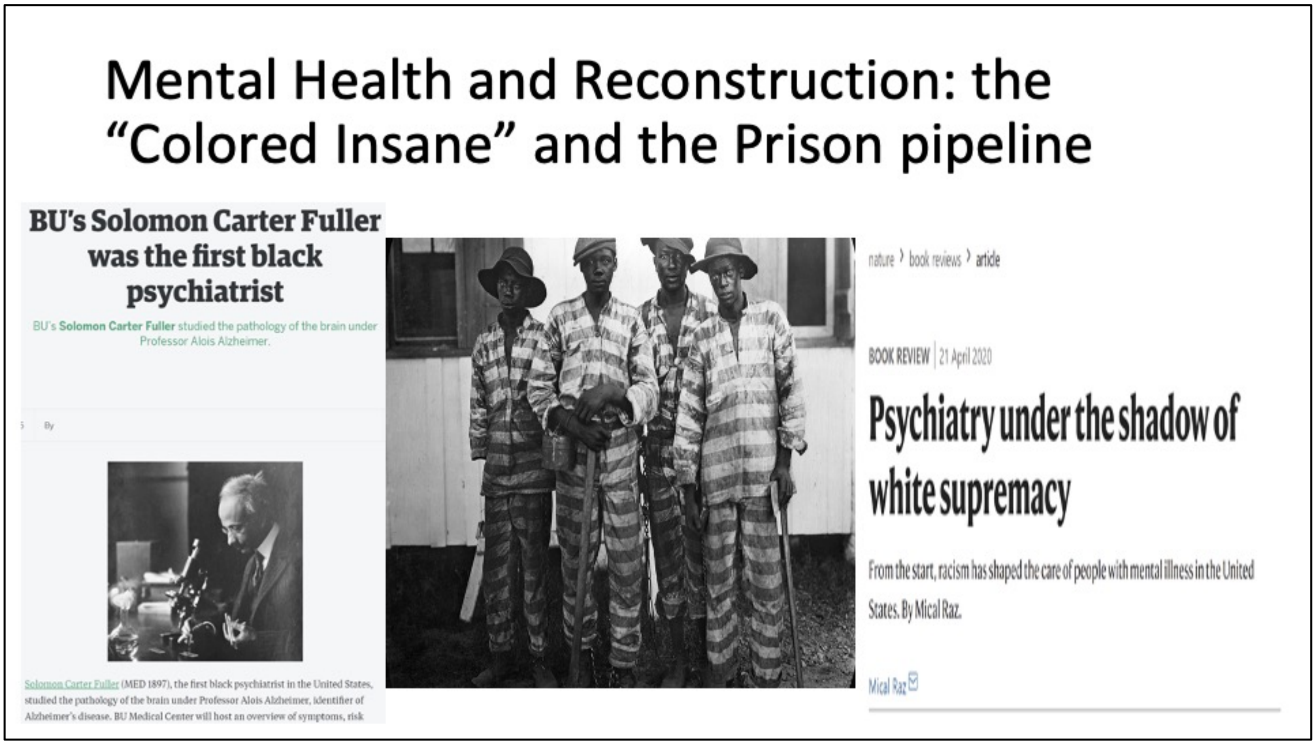

Freed blacks not working could be arrested for vagrancy or loitering, determined by the court as "insane" from historical data from psychology/psychiatry and forced into labor camps (14th amendment clause of the prison system as removing civil rights)

Hansan, J.E. (2011). Jim Crow laws and racial segregation. *Social Welfare History Project.* Retrieved [December 3 2024] from https://socialwelfare.library.vcu.edu/eras/civil-war-reconstruction/jim-crow-laws-andracial-segregation/
Raz, M. (2020, April 21). *Psychiatry under the shadow of White Supremacy*. Nature News. https://www.nature.com/articles/d41586-020-01126-w

- Zandonella, C. (2021, January 5). *Judith Weisenfeld explores psychiatry, Race and religion in the post-Civil War era*. Princeton University. https://www.princeton.edu/news/2021/01/05/judith-weisenfeld-explores-psychiatry-race-and-religion-post-civil-war-era

- Roberts, K., & Bowman, B. (2017, February 6). *Exploiting black labor after the abolition of slavery*. The Conversation. https://theconversation.com/exploiting-black-labor-after-the-abolition-of-slavery-72482

- *Bu’s Solomon Carter Fuller was the first black psychiatrist*. Boston University. (2005, December 6). https://www.bu.edu/articles/2005/bus-solomon-carter-fuller-was-the-first-black-psychiatrist/

Lynchings/Massacres

- Triggered by a trivial/stated strife- whistle, complaint, LWB
- Limit/control economic growth and conflict
- Rallied the community in support, fear, and supremist thought
- Terrorize/traumatize Black community

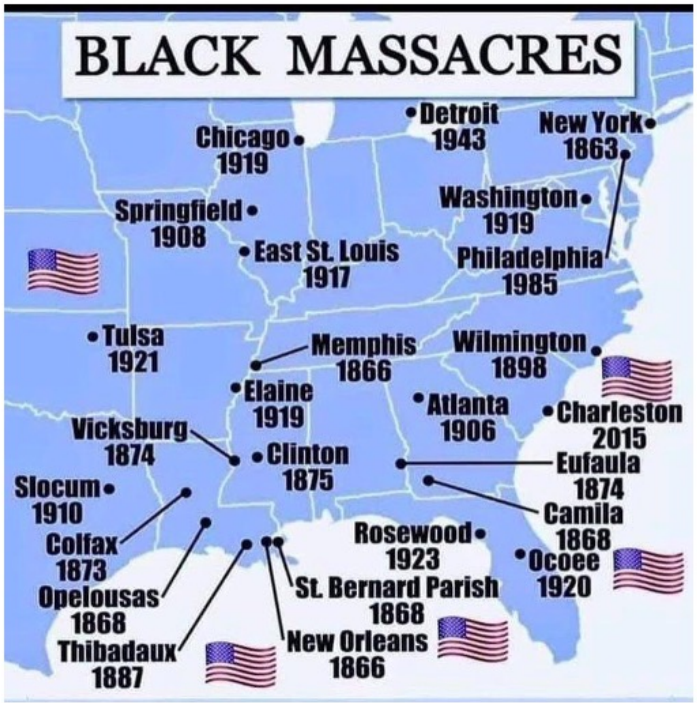

- Sinclair, B. (2022, April 10). *Who is black (black american)*. Medium. https://medium.com/afrosapiophile/who-is-black-american-59d54dac95ae

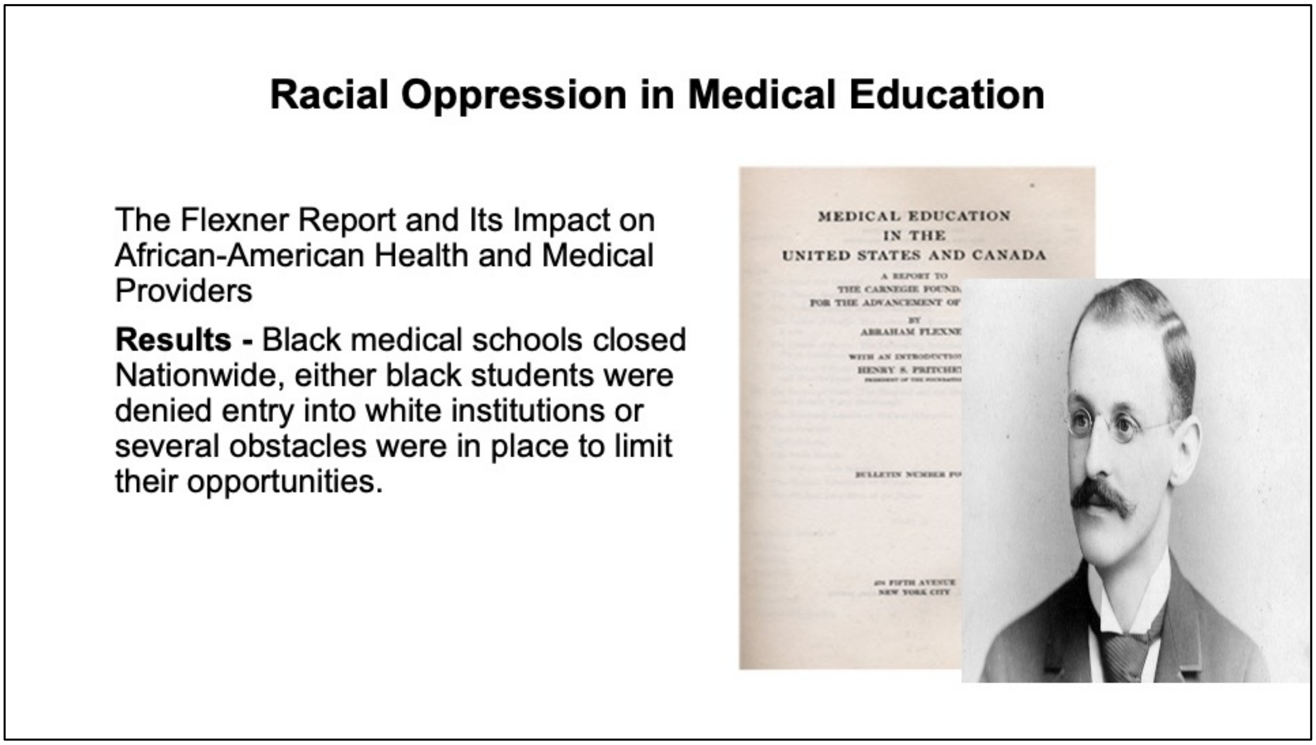

In response, missionary groups established medical schools, as did black physicians, who developed independent schools. In the late nineteenth century, as many as 12 black medical schools existed. There were nine black medical colleges at the time of Flexner's survey and only seven when he wrote his report in 1910; they included Flint in New Orleans, Leonard in Raleigh, and the Knoxville, Memphis, and Louisville schools. Given the lack of

resources and standards, they were under scrutiny for reform [49] (Table 1.1). When Flexner's report was released in 1910, he recommended closure of all but two of the seven black medical colleges (Howard University Medical Department and Meharry Medical Department of Central Tennessee College). He offered no mechanism for population-based needs assessment to develop a workforce to serve

the nearly 10 million black Americans living

- Medical University of South Carolina. (n.d.). *Flexner Report*. Flexner Report: 1913 State Takeover of MCSSC. https://waring.library.musc.edu/exhibits/SurvivingFlexner/?pg=Report

The Eugenics Movement and Mental Health

The American eugenics movement began in the late 19th century and continued as late as the 1940s. The movement centered around a goal to eliminate undesirable genetic traits in order to improve the human species.

- Laws legalized forced sterilizations of supposably “unfit’ people, >85% Black
- People with mental or physical defects and people of color were considered unfit
- Planned Parenthood was created by Margaret Sanger, a eugenicist who believed in selective breeding to achieve more “desirable traits”

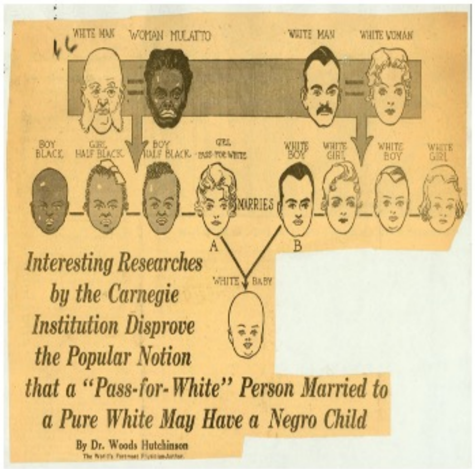

- Schambra, W. A. (2013, Summer). *Philanthropy’s original sin*. The New Atlantis. https://www.thenewatlantis.com/publications/philanthropys-original-sin

Pre-1910 Use of Substance in Society

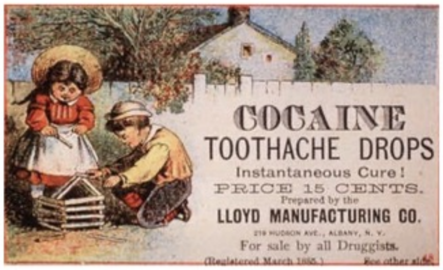

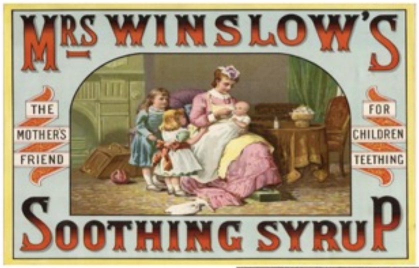

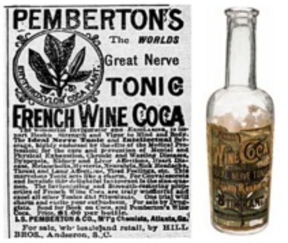

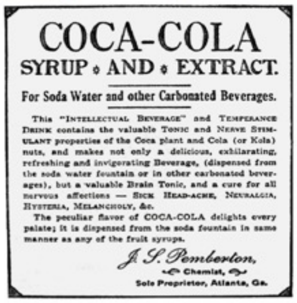

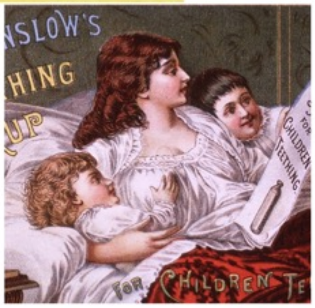

RUSH

Image Sourcing:
https://www.pharmacytimes.com/view/vintage-pharmacy-ad-promoted-cocaine-toothache-drops
https://www.aamc.org/news/grave-errors-spooky-cures-and-creepy-medical-missteps-past
https://chattanoogahistory.com/cocacolabottling
https://exhibits.library.stonybrook.edu/s/digital-projects/item/624
https://www.dailymail.co.uk/news/article-5036385/The-dark-history-world-s-popular-brands.html

What Changed? Early 20th Century: Law and Immigration

1914 Harrison Narcotics Tax Act

- Regulated manufacture and distribution of prescription opioids
- Licensing of pharmacists and physicians
- Permitted dispensing opioids to a patient in the course of the physician's professional practice only – no more OTC narcotics

1919-1920 Supreme Court Cases

- Criminalized prescribing/dispensing of opioids for individuals with opioid use disorders

1919 – 1935

- 25,000 physicians indicted for Harrison Act violations
- All morphine maintenance clinics closed

Medical treatment for substance use disorders disappears

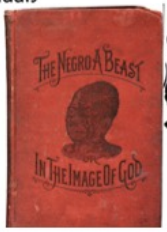

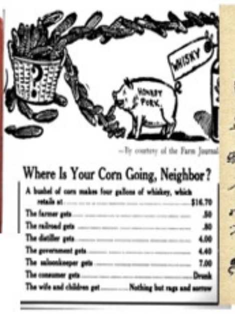

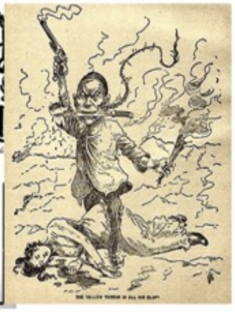

RUSH

Racialized, marginalized and criminalized those in our society that we didn't think were worthy.

Image Sourcing:
https://www.si.edu/object/nmaahc_2010.13
https://understandingrace.org/history/government/anti-chinese-laws-and-the-spanish-american-war-1880-1900/
https://quod.lib.umich.edu/b/bhl/x-bl000402/BL000402?chaperone=S-BHL-X-BL000402+BL000402;size=50;view=entry

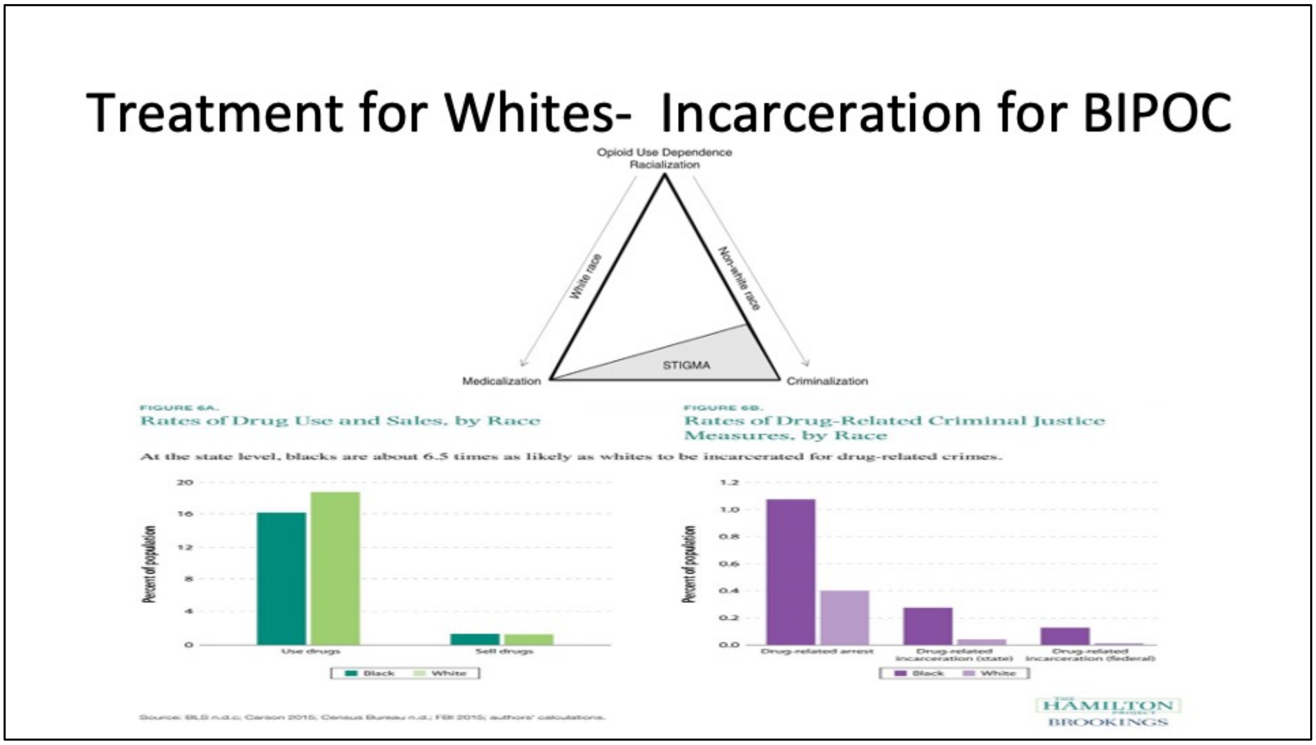

Mendoza, S., Hatcher, A.E., Hansen, H. (2019). Race, Stigma, and Addiction. In: Avery, J., Avery, J. (eds) The Stigma of Addiction. Springer, Cham. https://doi.org/10.1007/978-3-030-02580-9_8

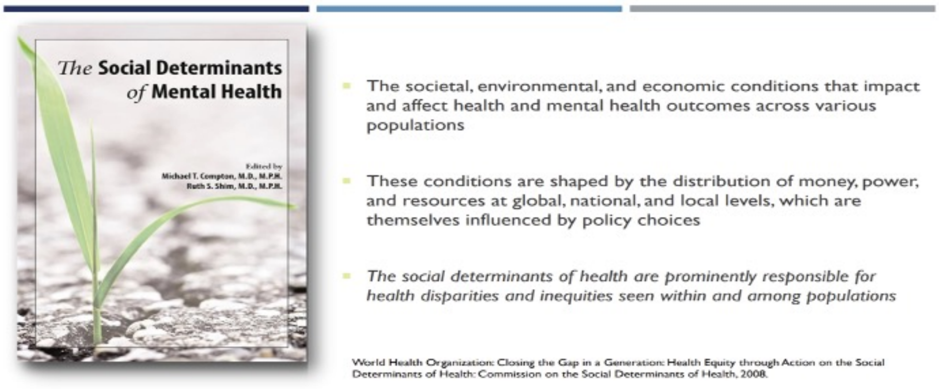

- Race is a social construct It is not clearly categorized
- We use race to confirm assumptions/prejudices/biases about our patients and clients
- Race is a rough proxy for culture, genetics, and socioeconomic status

- Shim, R. (n.d.). *Addressing discrimination and racism in health care*. CCSI. https://ccsi.org/CCSI/media/pdfs/Racism-and-Discrimination-in-Health-Care.pdf

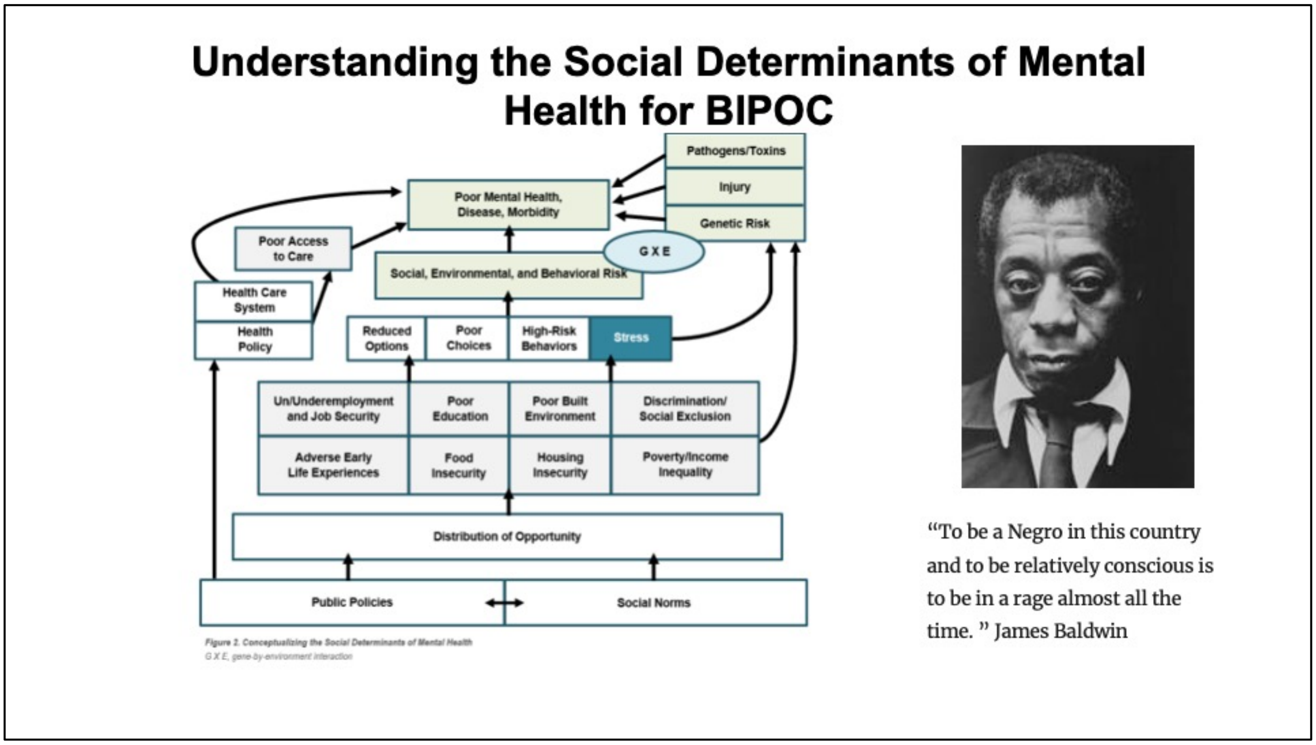

"To be a Negro in this country and to be relatively conscious is to be in a rage almost all the time. "

Fettes, D. L., Sklar, M., Green, A. E., Sandhu, A., Hurlburt, M. S., & Aarons, G. A. (2021). Racial and Ethnic Differences in Depressive Profiles of Child Welfare-Involved Families Receiving Home Visitation Services. *Psychiatric services (Washington, D.C.)*, *72*(5), 539–545. https://doi.org/10.1176/appi.ps.201900256

Alegría, Margarita et al. "Social Determinants of Mental Health: Where We Are and Where We Need to Go." *Current psychiatry reports* vol. 20,11 95. 17 Sep. 2018, doi:10.1007/s11920-018-0969-9
https://www.choosingtherapy.com/racism-mental-health/

Compton, MT, Shim, RS: The social determinants of mental health. Focus 2015: 13 (4): 419-425.

Alegría, M., NeMoyer, A., Falgàs Bagué, I., Wang, Y., & Alvarez, K. (2018). Social Determinants of Mental Health: Where We Are and Where We Need to Go. *Current psychiatry reports*, *20*(11), 95. https://doi.org/10.1007/s11920-018-0969-9

What's the result?

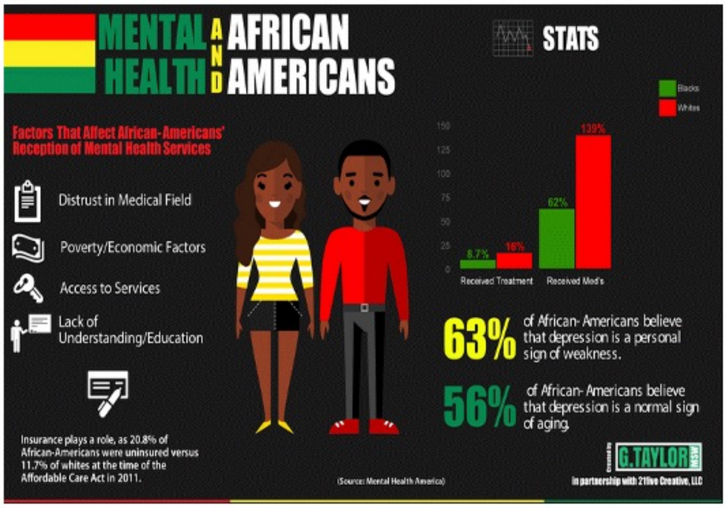

Because of the way that we developed in our history of Psychiatry and it's views of BIPOC, the way we currently assess, diagnose, treat BIPOC groups

- (2017, March 8). *From "birth of a nation" to "fistfight:" how the media stereotypes black men*. VOX ATL. https://voxatl.org/media-stereotypes-black-men/
- Image Sourcing: Mental Health America

Incarceration Associated With Homelessness, Mental Disorder, and Co-occurring Substance Abuse
Dale E. McNiel, Renée L. Binder, and Jo C. Robinson
Psychiatric Services 2005 56:7, 840-846

Jail Incarceration, Homelessness, and Mental Health: A National Study
Greg A. Greenberg, Ph.D. and Robert A. Rosenheck, M.D

Judicial interventions for mental health

Despite ~2,700 Crisis intervention team (CIT) programs nationwide and decades of operation, limited data is available to support CIT's effectiveness in reducing lethal outcomes in encounters with law enforcement.

Available Data on Police Encounters Estimates..

At least 25% of fatal police encounters involve persons with mental illness

76% of individuals killed in police encounters have had previous mental health treatment

Black peoples' fatality rate is 2.8 times that of their White counterparts in police encounters

More comprehensive data is needed to fully understand the impact of racism and mental illness on outcomes of lethal use of force by law enforcement officers.

- Shadravan, S. M., Edwards, M. L., & Vinson, S. Y. (2021). Dying at the intersections: Police-involved killings of black people with mental illness. *Psychiatric Services*, *72*(6), 623–625. https://doi.org/10.1176/appi.ps.202000942

DeGue, S., Fowler, K. A., & Calkins, C. (2016). Deaths Due to Use of Lethal Force by Law Enforcement: Findings From the National Violent Death Reporting System, 17 U.S. States, 2009-2012. *American journal of preventive medicine*, *51*(5 Suppl 3), S173–S187. https://doi.org/10.1016/j.amepre.2016.08.027

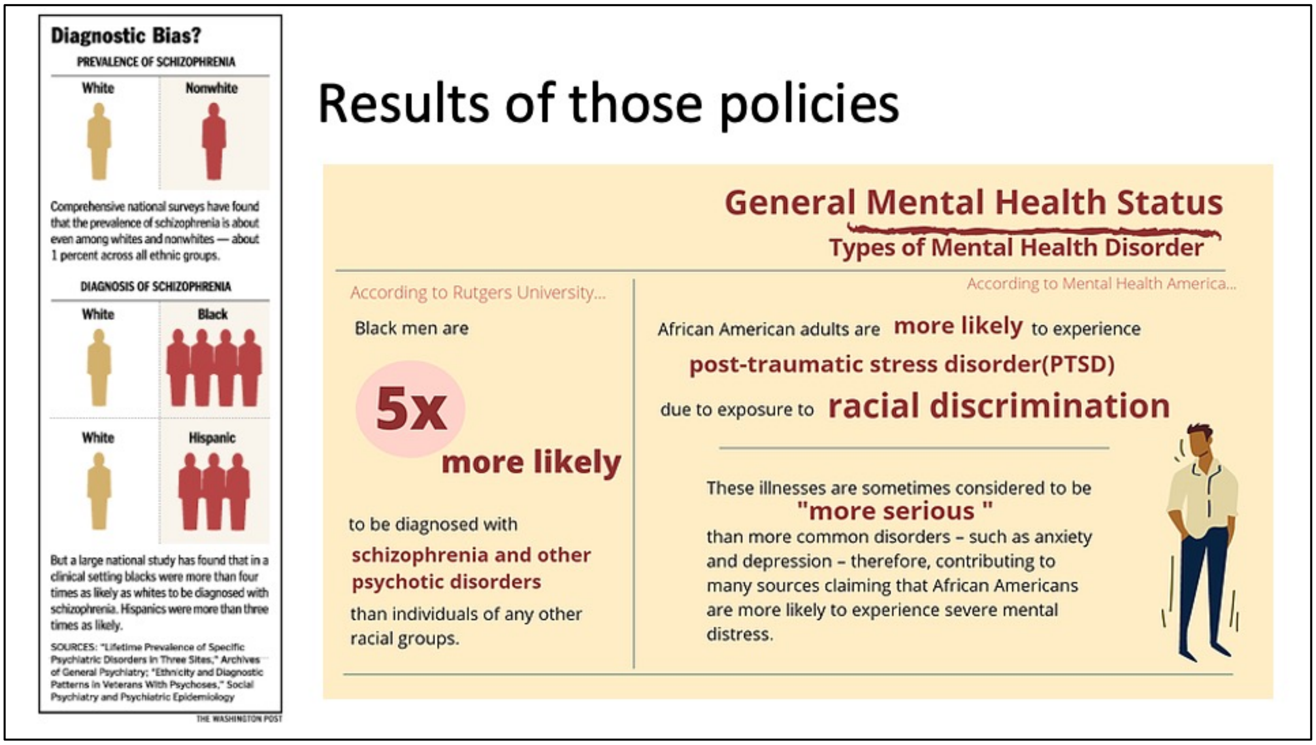

There was a slide that showed blacks were more likely diagnosed with schizophrenia and Serious Mental Illness rather than less benign mental health issues- where'd it go?

- Vedantam, S. (2005, June 28). *Racial disparities found in pinpointing mental illness*. Washington Post. https://psychrights.org/articles/WashPostRacialDisparities.htm
- Detester Magazine. (n.d.). https://detester.org/publications/16mentailhealthinafricanamerican

Disparities in Mental Health Care

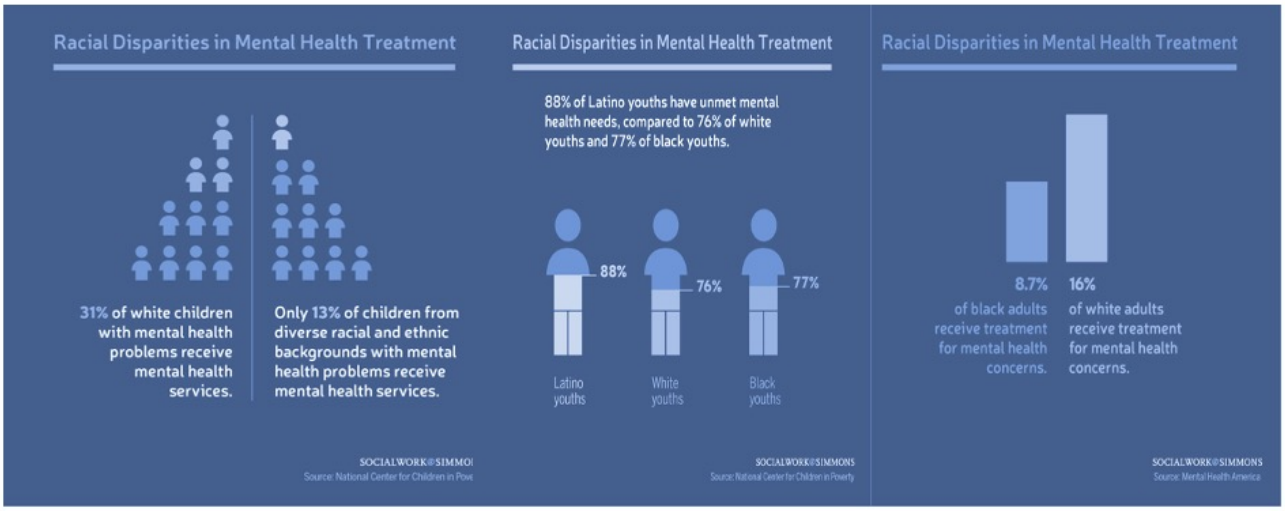

National Alliance on Mental Illness, 2017

National Center for Children in Poverty

https://online.simmons.edu/blog/racial-disparities-in-mental-health-treatment-text-only/#one

Juda, E. (2021, May 24). *Racial disparities in mental health treatment*. SC-UMT. https://online.simmons.edu/blog/racial-disparities-in-mental-health-treatment-text-only/#one

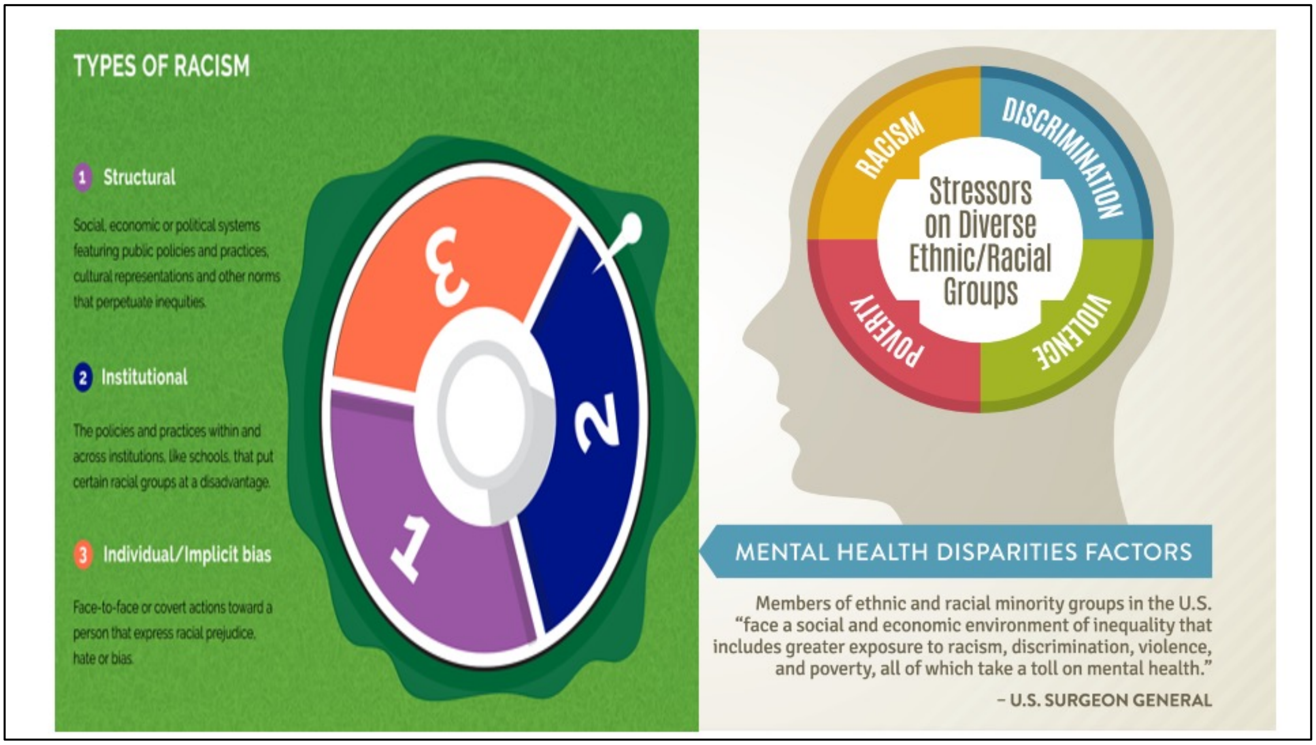

- Structural racism, defined by the Aspen Institute (5) as "a system in which public policies, institutional practices, cultural representations, and other norms work in various, often reinforcing ways to perpetuate racial group inequity," is the underlying mechanism that drives the unequal distribution of opportunities and advantages in society. Structural racism ties together mutually reinforcing social, economic, and legal systems that inequitably distribute power (e.g., wealth, rights, and education) (2).
- Roundtree, P. J. (2018, September). *Black Mental Health Matters*. TED Talk. https://www.ted.com/talks/phillip_j_roundtree_black_mental_health_matters?subtitle=en

Strong and Pervasive Evidence of Racial Inequalities POVERTY OUTCOMES Structural Racism By Keith Lawrence, Aspen Institute on Community Change and Terry Keleher, Applied Research Center at UC Berkeley For the Race and Public Policy Conference 2004

Institutional racism is defined as "discriminatory treatment, unfair policies and inequitable opportunities and impacts, based on race, produced and perpetuated by institutions (schools, mass media, etc.). Individuals within institutions take on the power of the institution when they act in ways that advantage and disadvantage people, based on race."

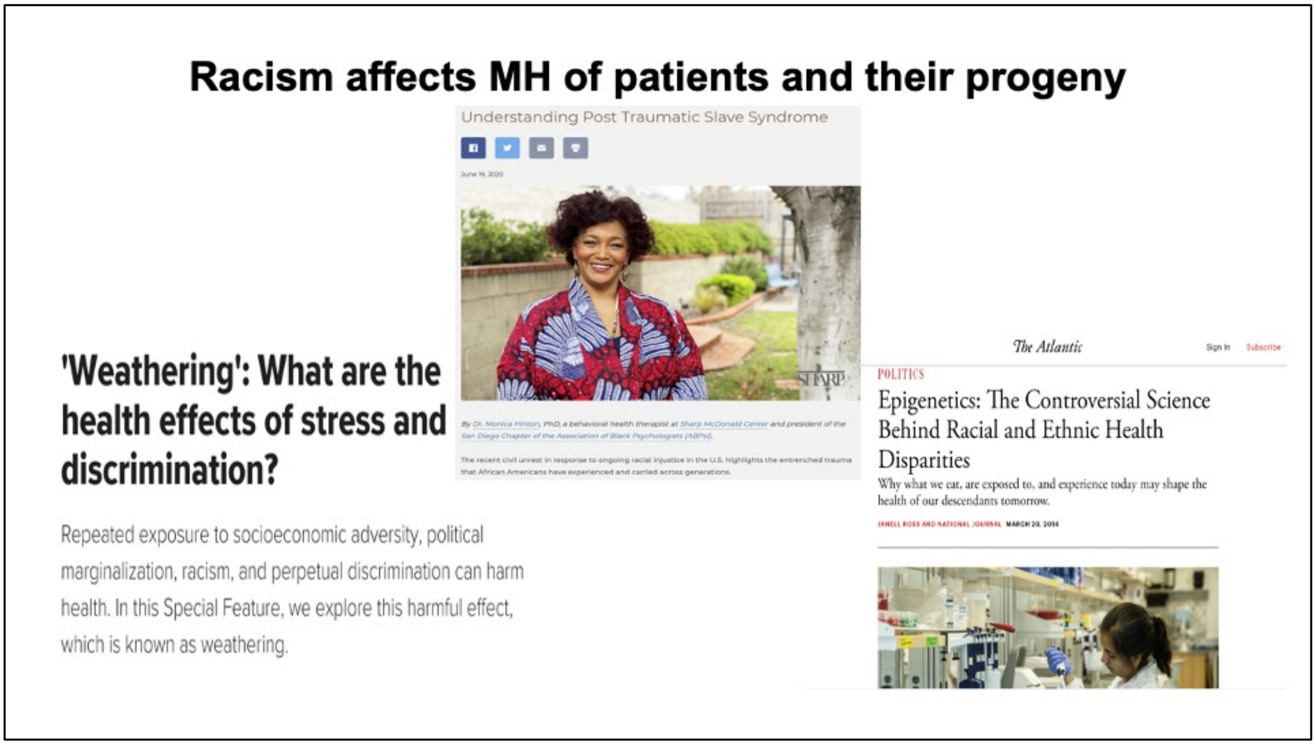

- Sandoiu, A. (2021, February 26). *“weathering”: The health effects of stress and discrimination*. Medical News Today. https://www.medicalnewstoday.com/articles/weathering-what-are-the-health-effects-of-stress-and-discrimination
- Hinton, M. (2020, June 19). *What is post traumatic slave syndrome?: Sharp Healthcare*. What Is Post Traumatic Slave Syndrome? | Sharp HealthCare. https://www.sharp.com/health-news/understanding-post-traumatic-slave-syndrome
- Ross, J. (2014, March 20). *Epigenetics: The controversial science behind racial and ethnic health disparities*. The Atlantic. https://www.theatlantic.com/politics/archive/2014/03/epigenetics-the-controversial-science-behind-racial-and-ethnic-health-disparities/430749/

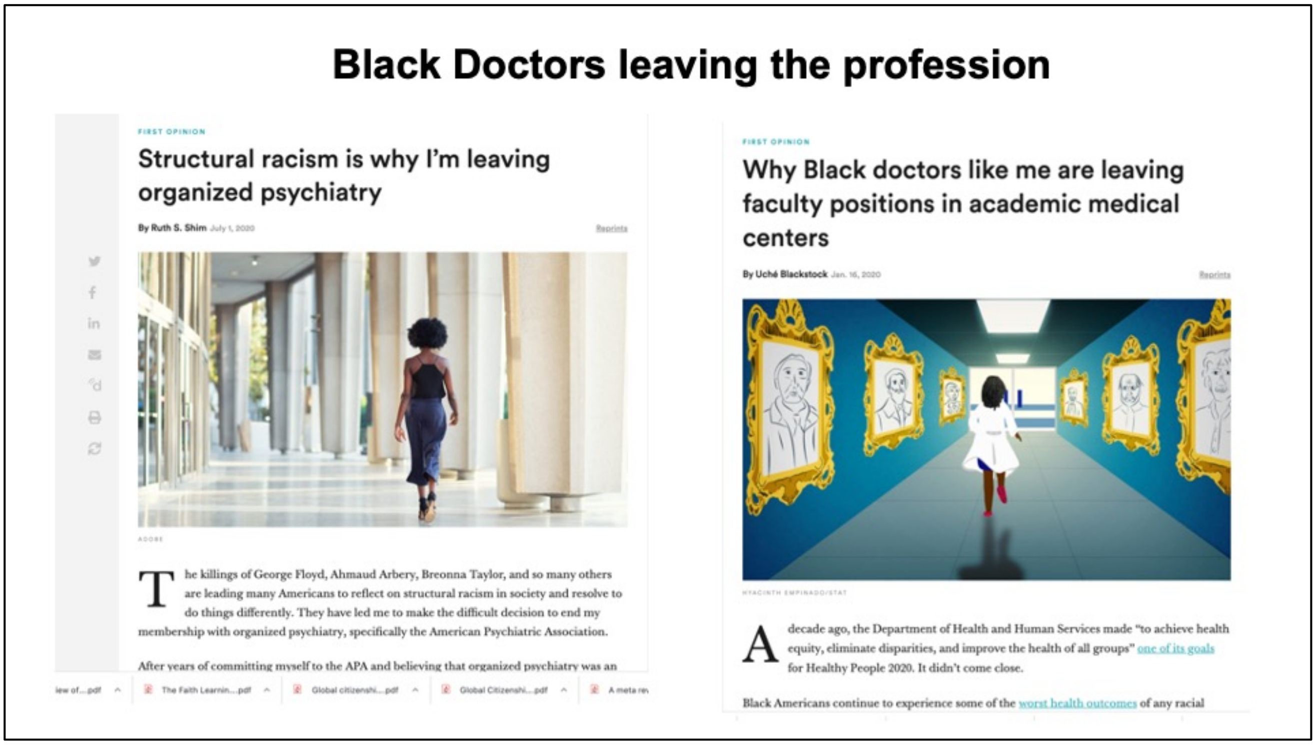

- Shim, R. S. (2020, July 1). *Structural racism is why I'm leaving organized psychiatry*. STAT. https://www.statnews.com/2020/07/01/structural-racism-is-why-im-leaving-organized-psychiatry/
- Blackstock, U. (2020, January 16). *Why black doctors like me are leaving faculty positions in Academic Medical Centers*. STAT. https://www.statnews.com/2020/01/16/black-doctors-leaving-faculty-positions-academic-medical-centers/

Attempts at Racial Equity

Implicit Bias Test- Harvard Implicit Bias Test

- Developed to test implicit bias and its affect on care
- Ongoing discussions as to application for change
    - Bias- admit- white guilt ; deny- dismiss test
    - not always result in patient outcome changes
- What results in Outcomes changes?
    - Operationalizing care trajectories
    - Cross Cutting testing for all
        - decrease likelihood of subjectives entering decision making
        - Genetics/epigenetics - nature-nurturing in Psychiatry
        - Psychiatry art is the subjective

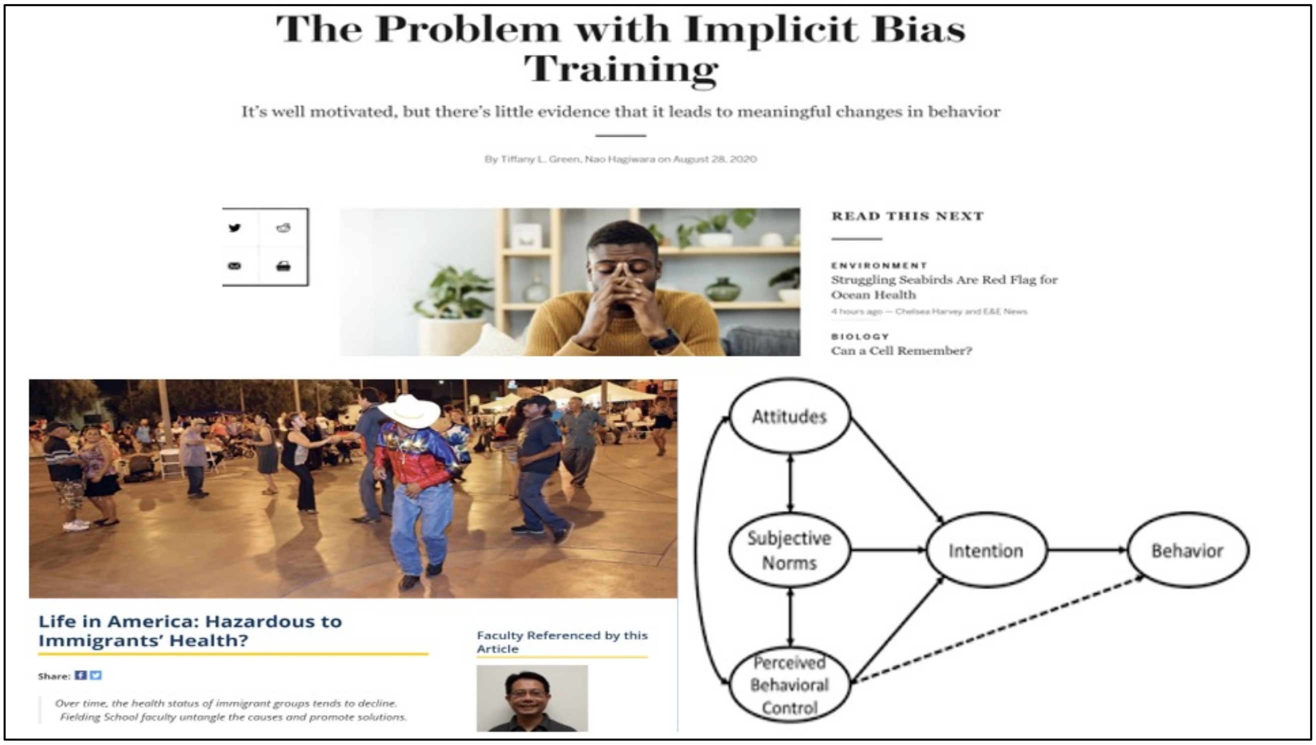

- Green, T. L., & Hagiwara, N. (2020, August 28). *The problem with implicit bias training*. Scientific American. https://www.scientificamerican.com/article/the-problem-with-implicit-bias-training/
- Gordon, D. (2014, December 2). *Life in america: Hazardous to immigrants' health?* UCLA. https://newsroom.ucla.edu/stories/life-in-america-hazardous-to-immigrants-health#:~:text=Public%20health%20experts%20have%20postulated,along%20with%20reduced%20physical%20activity.
- Sansom, R. (2021, March 9). *Theory of planned behavior*. Change Theories Collection. https://ascnhighered.org/ASCN/change_theories/collection/planned_behavior.html

https://ph.ucla.edu/news/magazine/2014/autumn/article/life-america-hazardous-immigrants-health

- Implicit Bias Test and discussion - https://www.youtube.com/watch?v=tkpUyB2xgTM

Where do we need to go? Truth/Reconciliation

AMERICAN PSYCHOLOGICAL ASSOCIATION

Date created: October 29, 2021

APA apologizes for longstanding contributions to systemic racism

Acknowledges failures, accepts responsibility, pledges change for psychology

WASHINGTON — As part of the nation's historic reckoning on racism, the American Psychological Association has apologized to communities of color for its role—and the role of the discipline of psychology—in contributing to systemic racism.

"For the first time, APA and American psychology are systematically and intentionally examining, acknowledging and charting a path forward to address their roles in perpetuating racism," said APA President Jennifer F. Kelly, PhD. "These resolutions are just the first steps in a long process of reconciliation and healing. This important work will set the path for us to make real change and guide the association and psychology moving forward."

- **APA issued formal apology in October 2021 for contributions to systemic racism**
  - APA Task Force on Strategies to Eradicate Racism
- **NIH Director issued a public apology in March 2021 to address funding disparity**
  - Launched an initiative called UNITE
    - Make public more data about the demographics of its staff and extramural grantees
    - Appoint a diversity officer at each of its 27 institutes and centers
    - Improve outreach about NIH's diversity training programs
- **AMA issued apology in 2008 for decades of racist policies toward Black doctors**

As of 2020 a few truths remain..

Truth: A study found 40% of first- and second-year medical students endorsed the belief that "black people's skin is thicker than white people's."

- Kaiser, J. (2021, March 1). *NIH director apologizes for 'structural racism,' pledges actions*. Science Insider. https://www.science.org/content/article/nih-director-apologizes-structural-racism-pledges-actions
- AMA apologies - NBCUniversal News Group. (2008, July 10). *Ama apologizes to Black Doctors for racism*. NBCNews.com. https://www.nbcnews.com/health/health-news/ama-apologizes-black-doctors-racism-flna1c9461472
- American Psychological Association. (2021, October 29). *Apa apologizes for longstanding contributions to systemic racism*. American Psychological Association. https://www.apa.org/news/press/releases/2021/10/apology-systemic-racism

- Truth 1 - Sabin, J. A. (2020, January 6). *How we fail black patients in pain*. AAMC. https://www.aamc.org/news-insights/how-we-fail-black-patients-pain

Addressing Racism in Psychiatry

- Anti-racist actions - acknowledging individual and structural racism through an examination of racist policies (Mensah, Ogbu-Nwobodo & Shim, 2021).
- Advocacy and attention are required to dismantle structural racism and to rebuild policies that support mental health equity.
- Centering racial equity as the standard of antiracism

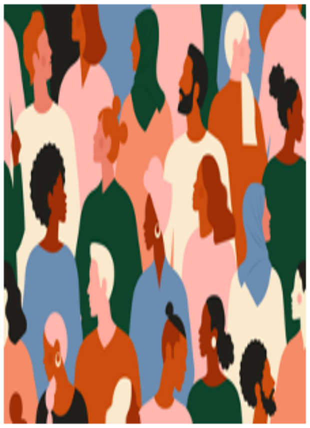

Racism and Mental Health Equity: History Repeating Itself
Michael Mensah, Lucy Ogbu-Nwobodo, and Ruth S. Shim
Psychiatric Services

Being an Anti-racist is Ethical

The American Medical Association's Principles of Medical Ethics states, "A physician shall recognize a responsibility to participate in activities contributing to the **improvement of the community and the betterment of public health."**

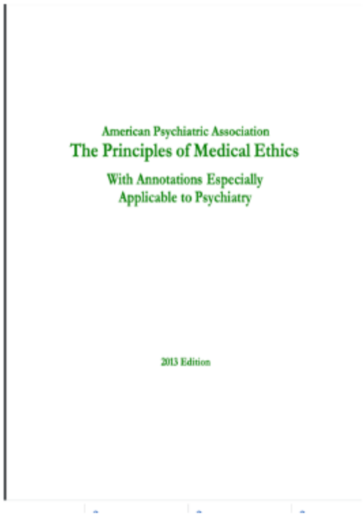
American Psychiatric Association
The Principles of Medical Ethics
With Annotations Especially Applicable to Psychiatry

2013 Edition

https://www.psychiatry.org/File%20Library/Psychiatrists/Practice/Ethics/principles-medical-ethics.pdf

American Psychiatric Association. (2013). Principles of medical ethics with annotations especially ... https://www.psychiatry.org/File%20Library/Psychiatrists/Practice/Ethics/principles-medical-ethics.pdf

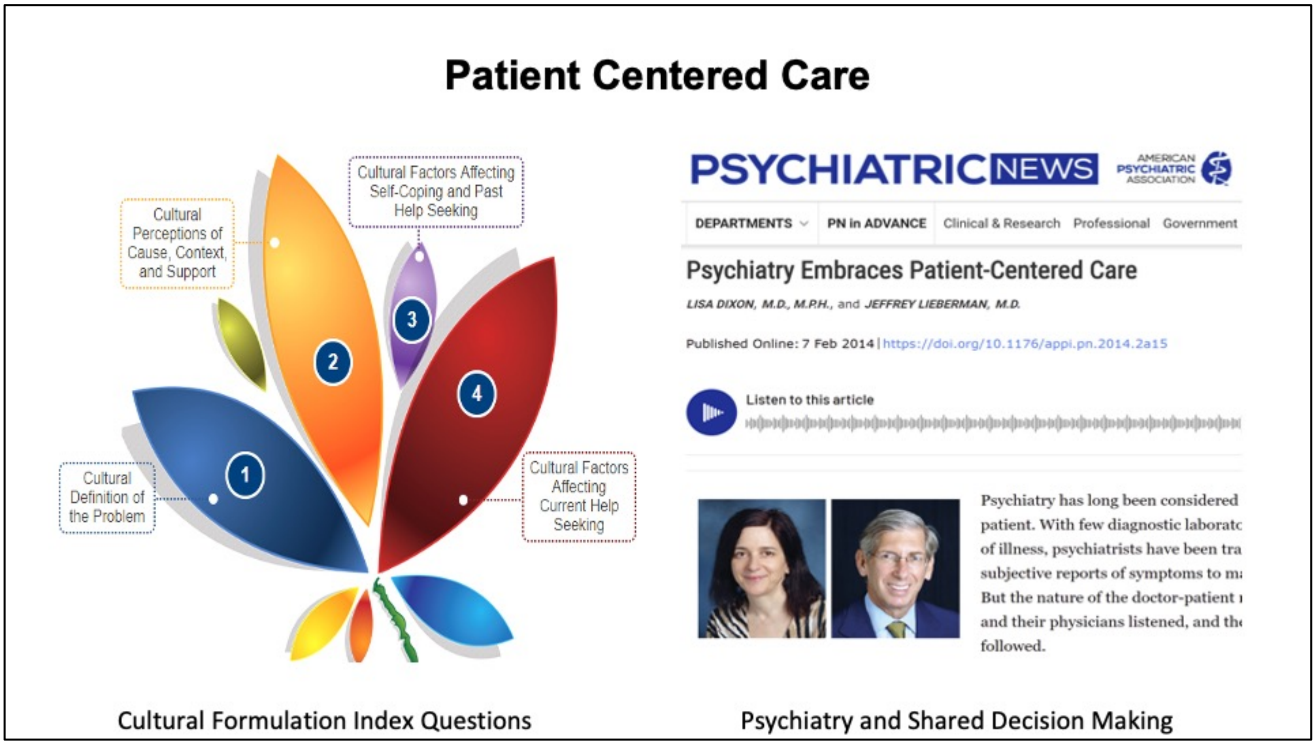

Improved outcomes in Mental Health care using the CFI

https://nyculturalcompetence.org/wp-content/uploads/2020/10/CFI.pdf

https://nyculturalcompetence.org/cfionlinemodule/

patient-centered care. This model of care places greater emphasis on the patient’s involvement in determining the goals of treatment that are meaningful to them and the nature of their care. Meaningful goals for patients generally go beyond symptoms to include quality of life, functioning, and a sense of hope and self-efficacy. Patient-centered care isn’t just about putting the patient at the center of the care equation. Rather, it shifts the balance of authority and responsibility of the doctor-patient relationship and incorporates shared decision making (SDM) between the clinician and the patient, particularly when it comes to treatment. SDM is defined as “a collaborative process that allows patients and their providers to make health care decisions together, taking into account the best scientific evidence available, as well as the patient’s values and preferences.” Practicing SDM requires that psychiatrists assess the patient’s interest in participating in decisions, providing information to them on the risks and benefits of specific treatments or approaches in an understandable format, and dialogue with patients about their choices. SDM does not mean that psychiatrists don’t make strong recommendations; rather, it means that those recommendations need to be reconciled with patients’ views and choices.

Dixon, L., & Lieberman, J. (2014). Psychiatry embraces patient-centered care. *Psychiatric News*, *49*(3), 1–1. https://doi.org/10.1176/appi.pn.2014.2a15

CFI online training module: Center of excellence for cultural competence. Center of Excellence for Cultural Competence. (2022, March 10). https://nyculturalcompetence.org/cfionlinemodule/

Table. Addressing issues in racism

Issue	Measurement
Professional wellness	Monitor burnout percentages of Black psychiatrists on an annual basis with implementation of anti-burnout studies.
Police and criminal justice reform	Infuse mental health professionals into culturally diverse safety and security institutions, providing guidance and education.
Visual symbols of racism	Monitor annually the number of racist symbols and names that remain as well as the number of changes and removals made.
Crucial and productive conversations about racism	Linking with W.R. Bion (*Victorian literature and culture*. 2019; 47[10]:167-186) becomes required reading for all mental health care professionals.
Examining racial blind spots in psychiatric practice	Observe the proportion of psychiatry grand rounds, medical education offerings, professional meetings, and professional development that address racial bias in psychiatric and medical decision-making.
Lifelong education on race in psychiatry	Active learning and experiences can be guided, measured, and encouraged through Performance in Practice Assessment modules that focus on race in all its dimensions.
Save the Children	Annual traumatic symptoms prevalence and resilience monitoring of children across cultures.
Intersectionality in psychiatry	Identify different groups to which people belong and start collecting data of cases of discrimination against them annually.
Racially based criminalization of substance abuse disorders	Annual measurement and tracking of racial disparities in drug-related arrests and sentencing as well as referrals to appropriate treatment for substance-related crime.
Racial lessons learned in rural psychiatry	Monitor the number of Black American psychiatrists practicing in rural areas and the supports offered for retention as well as safety.

- Moffic, S., & Bailey, R. (2020, August 11). *Dismantle racism in psychiatry & society*. Psychiatric Times. https://www.psychiatrictimes.com/view/dismantle-racism-in-psychiatry-society

Interactive Case Studies

a. Mother and 15 y/o teen boy arrive 20 minutes late for Peds Psych intake d/t transportation and childcare issues. Told by secretary to reschedule for next month, mother visibly upset.

b. 21 y/o male with pressured speech, awake 2 days since admission, UDS +cannabis, +AH of mother who died of COVID-19, Dx: Paranoid Schizophrenia.

1. How are Individual, Institutional, and/or Structural issues at play in this case?
2. How would changes to systems and structural issues improve patient care and outcomes?
3. How can individual changes improve patient care outcomes?
4. How does it feels to identify and ameliorate these issues in this case?

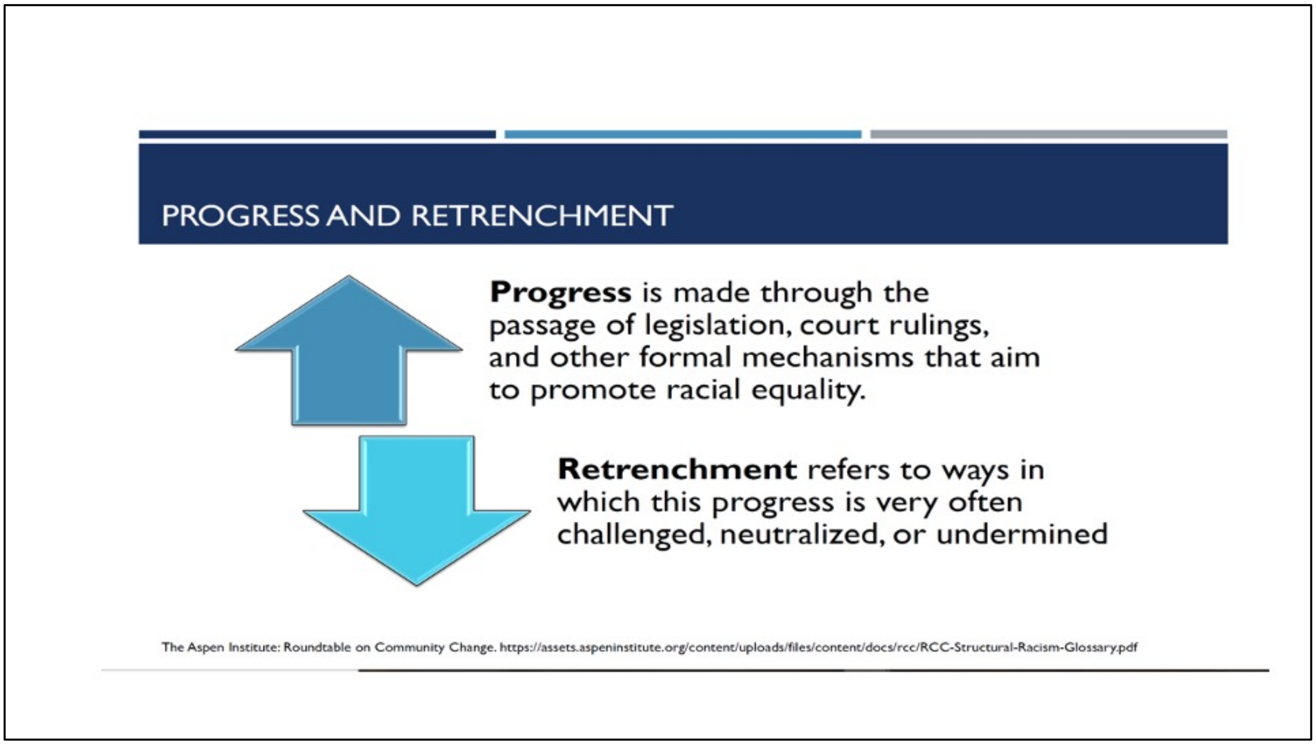

Reconstruction- 40 acres and a mule
Retrenchment- post- reconstruction Jim Crow, Homestead Act
Civil Rights Movement
Reagonomics- largest shift of wealth to the rich, “welfare queen,”
Obama elected president
Make America Great again
Biden elected, Stacey Abrams, 2 Demovratic Senators in Georgia

Removal of homosexuality as a DSM-3 diagnosis
Trans-rights
BLM

Image Source: Auspos, P., & Cabaj, M. (2014, September 24). *Complexity and Community Change: Managing adaptively to improve effectiveness*. The Aspen Institute. https://www.aspeninstitute.org/publications/complexity-community-change-managing-adaptively-improve-effectiveness/

Progress and Retrenchment

thebmj covid-19 Research Education News & Views

News

Black US doctor is fired after complaint about talk on racism in medicine

BMJ 2021 ; 372 doi: https://doi.org/10.1136/bmj.n116 (Published 13 January 2021)
Cite this as: *BMJ* 2021;372:n116

Article | Related content | Metrics | Responses

Jeanne Lenzer

Author affiliations

A doctor has been fired from her “dream job” as a small group facilitator at a medical school in California after she shared personal and historical incidents of racism during a talk with students.

Aysha Khoury, a 42 year old internist, was hired in July 2019 by Kaiser Permanente School of Medicine in Pasadena, which opened in 2020, does not charge fees, and encourages applications from students from diverse backgrounds. She was

P. S. Dr. Aysha Khoury is now a Professor at Howard University School of Medicine, an HBCU teaching this same content

- Lenzer, J. (2021). Black us doctor is fired after complaint about talk on racism in medicine. *BMJ*. https://doi.org/10.1136/bmj.n116

What can you as a medical student do?

Educate

Identify

Advocate

Conclusions

- Racism impacts physical/mental health in the US and the world
- Weathering and Generational Trauma issues perpetuate disparities
- The foundations of Psychiatry included individual, institutional, and structural racism
  - The results of this were predictable and current
- Truth and Reconciliation must mirror the foundations
  - individual, institution, and structural
- Behaviors must change, Attitudes we hope will follow
- Progress must overcome Retrenchment
- You are a change agent in this process!

Healthcare, education, judicial system, all must address this

Evaluative Reflection

Please include your thoughts, reflections on today's training and how it can make an impact on your care of patients during and after your rotation.

Once posted in the private chat, they'll be de-identified and used for our course evaluation process. Thanks.

References

Fettes, D. L., Sklar, M., Green, A. E., Sandhu, A., Hurlburt, M. S., & Aarons, G. A. (2021). Racial and Ethnic Differences in Depressive Profiles of Child Welfare-Involved Families Receiving Home Visitation Services. *Psychiatric services (Washington, D.C.)*, *72*(5), 539–545. https://doi.org/10.1176/appi.ps.201900256

Alegría, Margarita et al. "Social Determinants of Mental Health: Where We Are and Where We Need to Go." *Current psychiatry reports* vol. 20,11 95. 17 Sep. 2018, doi:10.1007/s11920-018-0969-9

https://www.choosingtherapy.com/racism-mental-health/

Compton, MT, Shim, RS: The social determinants of mental health. Focus 2015: 13 (4): 419-425.

Alegría, M., NeMoyer, A., Falgàs Bagué, I., Wang, Y., & Alvarez, K. (2018). Social Determinants of Mental Health: Where We Are and Where We Need to Go. *Current psychiatry reports*, *20*(11), 95. https://doi.org/10.1007/s11920-018-0969-9

North R. L. (2000). Benjamin Rush, MD: assassin or beloved healer?. *Proceedings (Baylor University. Medical Center)*, *13*(1), 45–49. https://doi.org/10.1080/08998280.2000.11927641

Sabshin M, Diesenhaus H, Wilkerson R: Dimensions of institutional racism in psychiatry. Am J Psychiatry 1970; 127:787–793 Bailey ZD, Krieger N, Agénor M, et al.: Structural racism and health inequities in the USA: evidence and interventions. Lancet 2017; 389:1453–1463

Incarceration Associated With Homelessness, Mental Disorder, and Co-occurring Substance Abuse
Dale E. McNiel, Renée L. Binder, and Jo C. Robinson
Psychiatric Services 2005 56:7, 840-846

Jail Incarceration, Homelessness, and Mental Health: A National Study
Greg A. Greenberg, Ph.D. and Robert A. Rosenheck, M.D.

Additional References + Resources:

1. Social Determinants of Health. https://health.gov/healthypeople/priority-areas/social-determinants-health (accessed 8 August 2024)
2. Rama Krishnan KR, Lateef O, Gabriel S, Crown S, Rumoro D, McCullough G, Ansell D. Stronger Together: Advancing Equity for All. Rush University Medical Center 2022.
3. West Side United shares plans to address health disparities in nine Chicago communities. https://www.rushu.rush.edu/news/west-side-united-shares-plans-address-health-disparities-nine-chicago-communities (accessed 14 February 2023)
4. Ahmad NJ, Shi M. The Need for Anti-Racism Training in Medical School Curricula. *Acad Med*. 2017;92:1073. doi: 10.1097/ACM.0000000000001806
5. Cartwright S. Diseases and Peculiarities of the Negro Race. De Bow's Review. 1853;XI.
6. Drapetomania - 2005 - Question of the Month - Jim Crow Museum. https://jimcrowmuseum.ferris.edu/question/2005/november.htm (accessed 8 August 2024)
7. Plous S, Williams T. Racial stereotypes from the days of American slavery: A continuing legacy[1]. *J Appl Soc Psychol*. 1995;25:795–817. doi: 10.1111/j.1559-1816.1995.tb01776.x
8. Hoffman KM, Trawalter S, Axt JR, *et al.* Racial bias in pain assessment and treatment recommendations, and false beliefs about biological differences between blacks and whites. *Proc Natl Acad Sci U S A*. 2016;113:4296–301. doi: 10.1073/pnas.1516047113
9. Gordon-Achebe K, Hairston DR, Miller S, *et al.* Origins of Racism in American Medicine and Psychiatry. In: Medlock MM, Shtasel D, Trinh N-HT, *et al.*, eds. *Racism and Psychiatry: Contemporary Issues and Interventions*. Cham: Springer International Publishing 2019:3–19.
10. American Psychological Association. Apology to People of Color for APA's Role in Promoting, Perpetuating, and Failing to Challenge Racism, Racial Discrimination, and Human Hierarchy in U.S. American Psychological Association 2021.
11. Shim RS. Dismantling Structural Racism in Psychiatry: A Path to Mental Health Equity. *Am J Psychiatry*. 2021;178:592–8. doi: 10.1176/appi.ajp.2021.21060558
12. DallaPiazza M, Padilla-Register M, Dwarakanath M, *et al.* Exploring Racism and Health: An Intensive Interactive Session for Medical Students. *MedEdPORTAL*. 2018;14:10783. doi: 10.15766/mep_2374-8265.10783
13. Hess L, Palermo A-G, Muller D. Addressing and Undoing Racism and Bias in the Medical School Learning and Work Environment. *Acad Med*. 2020;95:S44–50. doi: 10.1097/ACM.0000000000003706
14. White-Davis T, Edgoose J, Brown Speights JS, *et al.* Addressing Racism in Medical Education An Interactive Training Module. *Fam Med*. 2018;50:364–8. doi: 10.22454/FamMed.2018.875510
15. Hansen H, Braslow J, Rohrbaugh RM. From Cultural to Structural Competency—Training Psychiatry Residents to Act on Social Determinants of Health and Institutional Racism. *JAMA Psychiatry*. 2018;75:117–8. doi: 10.1001/jamapsychiatry.2017.3894
16. Pacheco N, Trinh N-H, Jahan A, *et al.* Dismantling Structural Racism in Psychiatric Residency Training: Nurturing a New Generation of Black, Indigenous, and People of Color (BIPOC) Psychiatrists. *Acad Psychiatry*. 2022;46:657–62. doi: 10.1007/s40596-022-01678-1
17. Shim RS. Structural racism is why I'm leaving organized psychiatry. STAT. 2020. https://www.statnews.com/2020/07/01/structural-racism-is-why-im-leaving-organized-psychiatry/ (accessed 8 August 2024)
18. O'Brien BC, Harris IB, Beckman TJ, *et al.* Standards for reporting qualitative research: a synthesis of recommendations. *Acad Med*. 2014;89:1245–51. doi: 10.1097/ACM.0000000000000388
19. Cook BL, Hou SS-Y, Lee-Tauler SY, *et al.* A Review of Mental Health and Mental Health Care Disparities Research: 2011-2014. *Med Care Res Rev*. 2019;76:683–710. doi: 10.1177/1077558718780592
20. Holden K, McGregor B, Thandi P, *et al.* Toward culturally centered integrative care for addressing mental health disparities among ethnic minorities. *Psychol Serv*. 2014;11:357–68. doi: 10.1037/a0038122
